# Longitudinal Analysis of Inflammatory Response to SARS-CoV-2 in the Upper Respiratory Tract Reveals an Association with Viral Load, Independent of Symptoms

**DOI:** 10.1007/s10875-021-01134-z

**Published:** 2021-09-28

**Authors:** Diem-Lan Vu, Paola Martinez-Murillo, Fiona Pigny, Maria Vono, Benjamin Meyer, Christiane S. Eberhardt, Sylvain Lemeille, Elodie Von Dach, Géraldine Blanchard-Rohner, Isabella Eckerle, Angela Huttner, Claire-Anne Siegrist, Laurent Kaiser, Arnaud M. Didierlaurent

**Affiliations:** 1 Division of Infectious Diseases, Geneva University Hospitals, Geneva, Switzerland; 2 Laboratory of Virology, Division of Laboratory Medicine, Geneva University Hospitals, Geneva, Switzerland; 3 University of Geneva Medical School, Geneva, Switzerland; 4 Department of Pathology and Immunology, Faculty of Medicine, Center of Vaccinology, University of Geneva, Geneva, Switzerland; 5 Unit of Immunology and Vaccinology, Geneva University Hospitals and Faculty of Medicine, Geneva, Switzerland; 6 Geneva Centre for Emerging Viral Diseases, Geneva University Hospitals, Geneva, Switzerland; 7 Department of Microbiology and Molecular Medicine, Faculty of Medicine, University of Geneva, Geneva, Switzerland

**Keywords:** SARS-CoV-2, Cytokine, Nasal wash, Symptoms

## Abstract

**Background:**

SARS-CoV-2 infection leads to high viral loads in the upper respiratory tract that may be determinant in virus dissemination. The extent of intranasal antiviral response in relation to symptoms is unknown. Understanding how local innate responses control virus is key in the development of therapeutic approaches.

**Methods:**

SARS-CoV-2-infected patients were enrolled in an observational study conducted at the Geneva University Hospitals, Switzerland, investigating virological and immunological characteristics. Nasal wash and serum specimens from a subset of patients were collected to measure viral load, IgA specific for the S1 domain of the spike protein, and a cytokine panel at different time points after infection; cytokine levels were analyzed in relation to symptoms.

**Results:**

Samples from 13 SARS-CoV-2-infected patients and six controls were analyzed. We found an increase in CXCL10 and IL-6, whose levels remained elevated for up to 3 weeks after symptom onset. SARS-CoV-2 infection also induced CCL2 and GM-CSF, suggesting local recruitment and activation of myeloid cells. Local cytokine levels correlated with viral load but not with serum cytokine levels, nor with specific symptoms, including anosmia. Some patients had S1-specific IgA in the nasal cavity while almost none had IgG.

**Conclusion:**

The nasal epithelium is an active site of cytokine response against SARS-CoV-2 that can last more than 2 weeks; in this mild COVID-19 cohort, anosmia was not associated with increases in any locally produced cytokines.

**Supplementary Information:**

The online version contains supplementary material available at 10.1007/s10875-021-01134-z.

## Introduction

SARS-CoV-2 replicates primarily in the upper respiratory tract, where it is detected at high concentrations before and within the first days after symptoms onset and for several days or even weeks, depending on disease severity and host immune status [1–3]. A mean duration of SARS-CoV-2 RNA shedding of 17 days has been reported, with no live virus detected beyond 9 days [4]. High viral load is also detected in asymptomatic patients [5–7]. ACE2, the main receptor of SARS-CoV-2, is expressed specifically within the motile cilia of upper airway epithelial cells [6]; expression gradually decreases when moving to the lower airways [8, 9], which may explain why viral load is generally higher in the nose than in the oral cavity or lungs [10]. Severe disease is thought to be due to a migration of the infection to the lungs and a dysregulated innate immune response leading to an excessive inflammatory reactions causing tissue damage and, ultimately, multi-organ failure [11, 12]. Efficient early control of viral replication in the upper respiratory tract could hamper dissemination to the lower tract and thus effect a more rapid resolution of infection.

Despite viral tropism for the upper respiratory tract, rhinitis is not often associated with COVID-19 [13], suggesting a limited inflammatory response at this site. The fact that asymptomatic infections are commonly observed also argues against symptoms driven by the local inflammatory response. In contrast, anosmia (usually combined with ageusia) is frequently reported, in particular by patients with mild disease and in the absence of other local symptoms, and can persist after resolution of other symptoms [13]. The mechanism involved in loss of olfactory functions is largely unknown and may be the consequence of a SARS-CoV-2-specific local inflammatory response [14].

A better understanding of the determinants that may effectively control the early replication of the virus in the nose may help defining strategies to rapidly reduce viral load and therefore potentially limit transmission and disease complications. As for many respiratory infections, evaluation of the mucosal as well as the systemic response have shown the disconnection between them [15, 16]. Although, the nature of the excessive inflammatory response associated with severe disease is well described in blood and lungs [11, 17–19], only a few studies have assessed the early local response at the primary site of infection in the upper respiratory tract [20–26]. These studies focus mainly on severe disease underestimating mild disease that represents the majority of infected patients and therefore the main source of transmission. SARS-CoV-2 induces a robust antiviral innate response, characterized by an increase in gene transcripts encoding interferons, interferon-stimulated proteins, and pro-inflammatory cytokines and reduced expression of genes involved in metabolic pathways. To date, neither the kinetics of local innate responses nor their relationship with viral shedding and symptom burden have been described. It is also unclear whether a robust local response may prevent infection or limit transmission. We report a longitudinal analysis of local and blood cytokine responses, viral load, antibody response, and symptom burden in patients with mild COVID-19.

## Methods

### Study Design, Setting, and Population

This prospective observational cohort study was nested within “Understanding Covid,” an ongoing, single-center prospective observational study conducted at the Geneva University Hospitals (HUG), Switzerland, to examine early virological and early and late immunological responses among SARS-CoV-2-infected adults and children, with inclusion no more than two days after diagnosis, and their household contacts. The study was approved by the Geneva Cantonal Ethics Commission (2020-00516); written informed consent is required. Further details on the Understanding Covid study are available in the online supplement. For this nested study, additional inclusion criteria were a positive RT-PCR test at the visits that were predefined in the protocol, age ≥ 18 years and the ability to undergo a nasal wash (NW). Visits were performed by house call unless patients were hospitalized and took place from inclusion until symptom resolution, with a maximum of 4 visits roughly 7 days apart. Symptoms were reported by patients at time of inclusion and at each visit. The following symptoms were solicited: fatigue, fever, subjective fever, chills, headache, myalgia (systemic); difficulty breathing, cough, sore throat (respiratory); anosmia and ageusia. Additionally, six healthy controls without symptoms and with negative SARS-CoV-2 PCR and serology testing were included for one-time NW and serum collection.

### Nasal Wash

Nasal wash was performed according to the institutional protocol [27, 28] and adapted from previous publications [29, 30]. Briefly, 3 ml of NaCl 0.9% are injected in the nose for NW and regurgitated. 2 ml are then retrieved and mixed with 1 ml of DMEM with 2 × protease inhibitor (Thermo scientific, ref 78437) for protein preservation. A concomitant oropharyngeal swab was also collected as part of the main study.

### SARS-CoV-2 RNA Quantification

Nucleic acids from NW and oropharyngeal samples were extracted individually from 400 µL of each specimen, spiked with 20 µL of standardized canine distemper virus as internal control [31], using the NucliSens eMAG extraction (BioMérieux, France) kit, according to the manufacturer’s instructions, and eluted in 50 µL. Quantitative real-time polymerase chain reaction (qRT-PCR) assays were performed using the one-step Eurogentec RT-PCR Kit (Qiagen, Hombrechtikon, Switzerland) in a QuantStudio 5 instrument (Applied Biosystems) and SARS-CoV-2 RNA was quantified with the Charité qRT-PCR protocol [32] using in vitro transcribed RNA for quantification [33] and reported as number of copies/ml of NW collected.

### Cytokine Measurement

A Luminex assay (Magnetic Luminex assay, R&D Systems) was used to calculate the concentration of 23 markers in cryopreserved plasma and NW. For plasma, assays were performed according to the supplier’s instructions. For NW, a last incubation step of 20 min with PFA 4% (followed by 4 washes) was added before reading to inactivate any remaining live virus. Briefly, beads conjugated to the analyte-specific capture antibodies, samples, controls, and standards were incubated at room temperature for 2 h. Biotinylated detector antibodies and R-phycoerythrin-conjugated streptavidin were subsequently added. The mean fluorescence intensity of each marker was read on the Bio-Plex 200 array reader (Bio-Rad Laboratories) using the Luminex xMAP Technology (Luminex Corporation). Five-parameter logistic regression curve (Bio-Plex Manager 6.0) was used to calculate sample concentrations. List of markers tested: CD40L, GM-CSF, Granzyme B, IFN-alpha, IFN-gamma, IL-1-beta, IL-1-alpha, IL-1Ra, IL-2, IL-4, IL-6, IL-8, IL-10, IL-12p70, IL-13, IL-17A, IL-33, CXCL10, CCL2, CCL3, CCL4, PD-L1, and TNF-alpha. Samples whose measurement was below the detection limit were assigned a value corresponding to 50% of the last standard dilution value.

### Antibody Characterization by ELISA

IgG and IgA antibodies directed against the S1 domain of the spike protein of SARS-CoV-2 were determined using a commercially available kit (Euroimmun AG, Lübeck, Germany, #EI 2606-9601 G and EI 2606-9601 A) according to the manufacturer's instructions. All nasal wash samples were tested at 1:2 dilutions, while for serum only samples outside linear range were diluted 1:5 or 1:10. In serum, S1-specific IgG and IgA were positive when > 0.8 OD ratio [34], while for nasal wash S1-specific IgA was positive when > 0.4 OD_450_ (highest value of the control group). Total IgA measurements in nasal wash were performed using a Thermo Fisher IgA human ELISA kit (pre-coated) and all samples were tested at 1:100 dilutions.

### Statistical Analysis

Categorical variables were described by counts and percentages. Continuous variables were expressed as median and interquartile range (IQR). Correlation analyses were performed using the Pearson test. Cytokine data were categorized by defined time interval post onset of symptoms and differences in intervals between patients and healthy controls were calculated using the Mann–Whitney test. Principal components analysis was performed in R version 3.6.2 using the prcomp function of the stats package. Data were standardized by the scale function in R. The first two principal components were retained since the first two eigen values were higher or close to 1. The first two components explained 75.45% and 12.11% of the variance, respectively. A bootstrap procedure was used to check the robustness of each retained principal component. Fifty-thousand re-sampling with replacement were done. For each re-sampling, the same PCA was conducted. The frequency of the number of retained components (eigen value > 1) over the 50,000 re-sampling was 100% for PC1 and 49.19% for PC2.

## Results

### Clinical Presentation and Viral Load

**Fig. 1:**
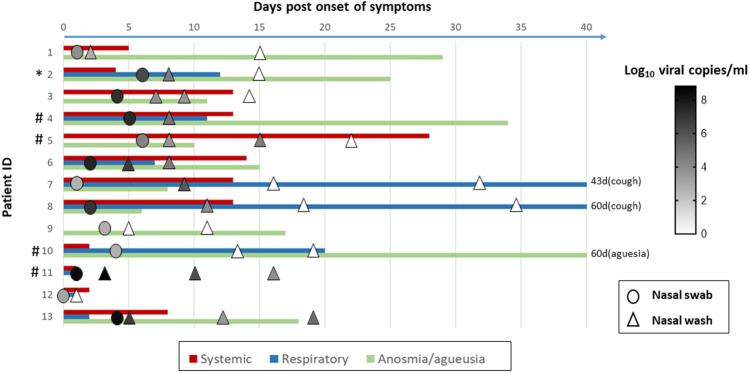
Longitudinal analysis of reported symptoms and viral loads in nasal wash of SARS-CoV-2 patients. The duration of systemic (headache, fever, shivering, myalgia, or fatigue), respiratory (sore throat or cough), and anosmia/ageusia are shown according to the first day of reported symptoms. Viral loads were measured by PCR in nasal swabs (circle) at time of diagnosis or in nasal washes (triangle) at different visits and levels are shown in the symbols as white (undetectable) to dark gray according to the heatmap shown in the figure. *Patient 2 reported a rhinitis for a duration of 11 days. # Patient 4, 5, 10, and 11 reported difficulty breathing for 10, 40 days, 4 days, and 1 day, respectively (not reported in the figure)

Thirteen SARS-CoV-2-infected patients and six controls were included. Patients’ median age was 35 years (mean 14.2); 12 had mild disease and one had severe disease requiring oxygen therapy (patient 3), as reflected in the CRP values and blood cell counts at time of diagnosis (Table 1). Figure 1 depicts timing of NW collection, viral load, and duration of symptoms for each patient by day post onset of symptoms (DPOS). Diagnosis was made at a median of 5 DPOS (range 1–8 DPOS), with median viral load at diagnosis of 1.9 × 10^6^ RNA copies/ml detected in nasopharyngeal specimens (Table 1).

**Table 1:** Patient characteristics

Pat ID	Age	Sex	DPOS at diagnosis	Routine NP VL (cp/ml sample)	OP VL V1 (cp/ml sample)	NW VL V1 (cp/ml sample)	CRP V1 (mg/l)	Lc V1 (G/l)	Lymphocytes V1 (G/l)	PMNs V1 (G/l)	Tc V1 (G/l)
1	30	M	1	7.98 × 10^3^	3.64 × 10^3^	7.62 × 10^2^	4	4.8	2.21	2.06	188
2	33	F	6	1.86 × 10^6^	ND	3.58 × 10^4^	2.5	6.1	2.28	3.32	241
3^a^	72	M	4	1.28 × 10^7^	8.46 × 10^2^	6.72 × 10^3^	173	10.2	1.33	7.74	257
4	40	F	5	2.39 × 10^7^	5.66 × 10^4^	6.35 × 10^4^	19.7	2.7	0.84	1.68	198
5	46	F	6	1.70 × 10^4^	2.83 × 10^5^	4.05 × 10^3^	0.6	2.7	1.13	1.14	283
6	28	M	2	3.61 × 10^7^	6.79 × 10^6^	5.42 × 10^6^	13.6	4.1	1.29	1.99	140
7	30	M	1	5.41 × 10^2^	5.56 × 10^4^	ND	NA	4.5	1.04	2.38	194
8^b^	32	F	2	9.73 × 10^6^	1.2 × 10^7^	NA	2.8	3.5	1.31	1.87	146
9	35	F	3	5.41 × 10^2^	ND	ND	0.4	4.4	1.71	2.19	127
10	35	F	4	4.92 × 10^3^	1.98 × 10^3^	8.93 × 10^4^	0.4	8.2	1.84	5.7	248
11	74	F	1	2.49 × 10^8^	2.61 × 10^7^	6.94 × 10^8^	3	9.7	5.92	3.2	168
12	55	M	0	1.36 × 10^3^	ND	ND	1.7	4.8	2.88	1.01	258
13	35	M	4	2.49 × 10^8^	4.63 × 10^6^	6.72 × 10^6^	15.7	6.1	1.2	4.43	182
Median	35	-	3	1.86 × 10^6^	5.56 × 10^4^	2.12 × 10^4^	2.9	4.8	1.33	2.2	194

*NP* nasopharyngeal, *VL* viral load, *V1* visit 1, *NW* nasal wash, *CRP* C-reactive protein, *Lc* leukocytes, *PMN* polymorphonuclear, *Tc* thrombocytes, *cp* copies, *ml* milliliter, *mg* milligrams, *G* giga, *l* liter, *NA* not available, *ND* not detected

^a^This patient was hospitalized for severe pneumonia

^b^As per protocol, no V1 nasal wash was available for patient contact 20-2

The first NW was performed within 8 days after onset of symptoms at time where patients still reported symptoms, followed by up to three samplings until resolution of acute symptoms, except for patient 11 who became quickly asymptomatic. At this first time point, SARS-CoV-2 could be detected in NW in 11/13 patients, with a median value of 3.6 × 10^4^ RNA copies/ml NW (IQR 5.4 × 10^6^). Viral load in NW was comparable to that measured in oropharyngeal swabs, suggesting a potential use as an alternative method for viral detection in those not at ease with swabs (Table 1). The virus was detected within 11 DPOS in 10/13 patients, but by 14 DPOS, most patients were negative and their symptoms had resolved, except for 3 patients who still had detectable viral RNA in the 3^rd^ week post symptom onset. Two patients (11, 13) became rapidly pauci or asymptomatic, while relatively high level of viruses was still measured in their NW. Overall, there was no association between the presence of virus in the nose and particular respiratory symptoms. Rhinitis was reported by only one patient. No other common respiratory viruses were detected in the samples.

### Local Cytokine Profile

**Fig. 2:**
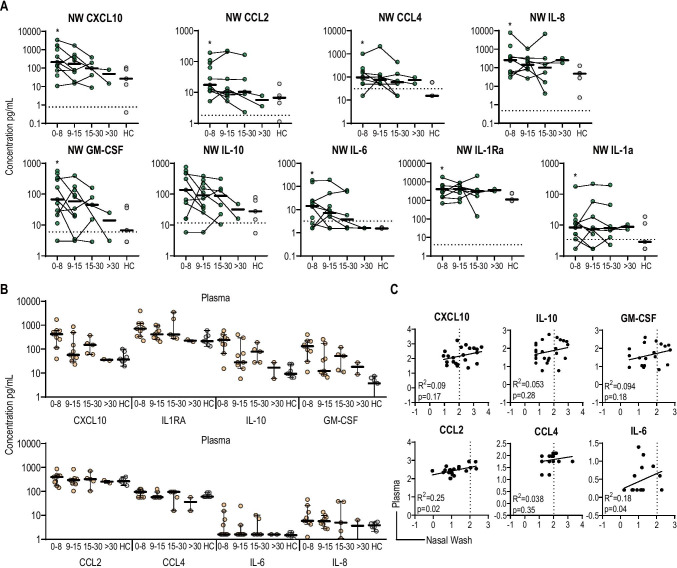
Cytokine profile in nasal wash and correlation with plasma cytokines. Cytokine concentrations in nasal wash (**A**) or plasma (**B**) were measured by multiplex Luminex assay. The data are presented according to four different intervals post onset of symptom: 0–8, 9–15, 15–30, and > 30. Circles represent the value for each participant and are connected to show the kinetics for each participant. Thick black lines represent the median for each interval; * represents significant difference (*p* < 0.05) between the respective interval with the healthy control group. **C** Correlation analysis between nasal wash and plasma cytokines. Dashed lines represent the limit of detection. *NW* nasal wash, *HC* healthy controls. Nasal wash samples are represented as green circles and plasma samples are represented as orange circles. Gray circles for the healthy control

We next assessed the innate response to SARS-CoV-2 in the upper respiratory tract by measuring the level of 23 pro-inflammatory cytokines in NW. Two cytokines (IL-6 and IL-10), IL-1 receptor alpha, four chemokines (CXCL10, CCL2, CCL3, and IL-8), and one stimulating factor (GM-CSF or CSF-2) were detectable, including in controls (Fig. 2a). Median concentrations of CXCL10, IL-6, and IL-10, hallmark cytokines circulating in blood during COVID-19 disease [35], reached relatively high levels in the first week (CXCL10: 214.4 [95%CI, 39.52 to 1680]; IL-6: 14.4 [95%CI, 1.6 to 155]; IL-10: 137.2 [95% CI, 16.10 to 576.4] in pg/ml) and decreased gradually to levels measured in healthy control after 2–3 weeks post onset of symptoms. IFN-alpha levels were under detection levels (< 1.5 pg/ml) for most participants (25/28). CCL2, a chemoattractant for monocytes, as well as IL-8, a chemoattractant for neutrophils, were also significantly increased within the first week in most patients (CCL2: 17.51, [95%CI, 11.04 to 110]; IL-8: 256.6, [95%CI, 46.19 to 1507] in pg/ml) suggesting that monocytes and neutrophils could be recruited locally during mild infection. Finally, GM-CSF, which favors differentiation of macrophages and antigen-presenting cells activation and therefore could play a role in the elimination of the virus [36], was more persistently found in the NW of infected individuals as compared to the healthy groups. We were not able to investigate the cellular composition of the NW because of the limited number of cells recovered in the samples.

We then assessed whether a similar pattern of cytokines was observed in plasma at the same time points in infected patients and healthy controls (Fig. 2b). The median concentrations of some cytokines were generally higher in NW than in plasma, in particular for IL-8 (NW: 256.6; plasma: 5.85 pg/ml) and IL-6 (NW: 14.4; blood: 1.6 pg/ml), and the increase in CCL2 (but not absolute level) was more significant locally. Interestingly, we did not find any correlations between the cytokine levels detected in NW and plasma (Fig. 2c), suggesting that measure of inflammation in blood does not reflect local (nasal) innate response at least in case of mild disease.

### Correlation Between Local Cytokine Responses and Viral Load

**Fig. 3:**
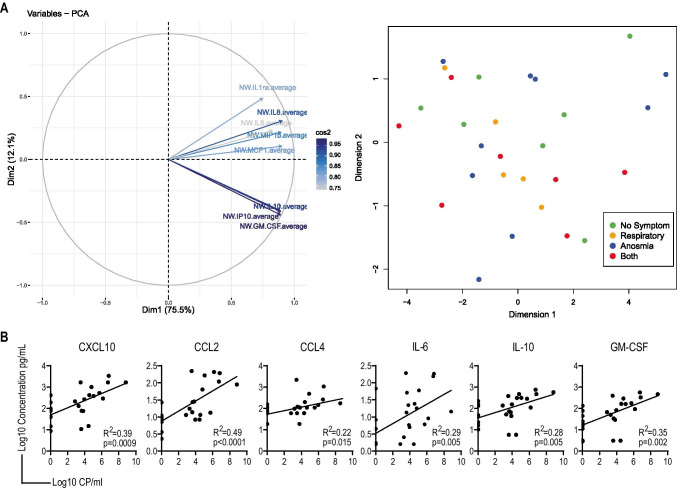
Correlation between local cytokines, symptoms, and viral load. **A** A principal component analysis was performed on cytokines measured in nasal wash, PC1, and PC2 are represented (right). The symptoms at time of sample collection were then overlaid on PC1 and PC2. No specific correlation was observed (left). **B** Correlation analysis between nasal wash cytokines and viral load measured in the same samples, both were log10 transformed to test the correlation; each panel shows *R*^2^ value and *p* value. *Cp* copies

A principal component analysis did not reveal any association between cytokine levels in NW and symptoms, including anosmia (Fig. 3a). There was also no correlation between cytokine levels and duration of symptoms as reported by participants. In contrast, all cytokine concentrations positively correlated with viral load (Fig. 3b), CCL2 and CXCL10 most closely (*R*^2^ = 0.49 and 0.39, respectively). This suggest that the cytokines detected in the NW are most likely a result of the local viral replication taking place in the upper respiratory tract.

### Local Antibody Levels

**Fig. 4:**
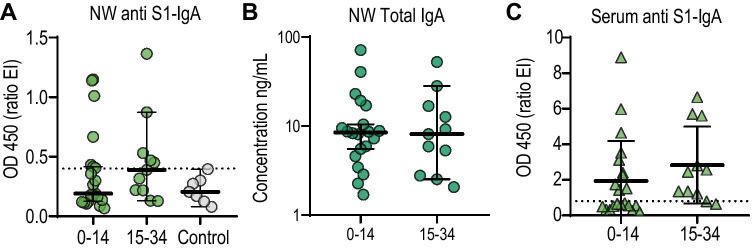
S1-specific IgA response in nasal wash and serum. S1-specific IgA (**A**) and total IgA (**B**) were measured by ELISA in nasal wash (NW). The dotted line represents the maximum value detected in the negative samples for S1-specific IgA (0.4). **C** S1-specific IgA OD ratio values in sera. The dotted line represents the detection level of the Euroimmune assay in serum (0.8). Nasal wash samples are represented as circles and serum samples are represented as triangles. Dark green for total IgA. Light green for S1-specific IgA. Gray for the control

In nasal wash, the levels of anti-S1 IgA above the detection level were 33.3% (7/21) of the participants in the first interval and 45.5% (5/11) in the second interval (Fig. 4A), while total IgA was detected for all the patients (Fig. 4B). The levels of anti-S1 IgG above the detection level were 0% (0/20) of the participants in the first interval and 18.2% (2/11) in the second interval (not shown). In sera, S1-specific IgA was more rapidly detected (48% positive, Fig. 4C) as compared to IgG (28% positive, not shown) within 14 DPOS. We also observed a weak correlation between serum and nasal wash S1-specific IgA (*R*^2^ = 0.38; *p* = 0.0002). Altogether, this suggest that S1-directed IgA and not IgG is detectable at the mucosal site in some individuals and correlates to some extent with S1-directed IgA antibodies in serum.

## Discussion

Cytokines were found locally in the upper respiratory during mild SARS-CoV-2 infection. Local cytokine responses persisted for up to two weeks after symptom onset; they correlated with viral load, irrespective of the time since symptom onset, but not with blood cytokine levels or specific symptoms. Our results are consistent with the transcriptional responses measured in nasopharyngeal specimens collected during SARS-CoV-2 screening, which show a positive correlation between gene expression and viral load [20, 22, 23, 25]. The presence of high levels of CXCL10 at the transcription and protein level, together with the detection of an interferon-stimulated gene signature [22, 23], definitively show that the upper respiratory tract is an important antiviral immune site during mild disease. Although an early IFN signal in the nose has been reported at the transcriptional level [21, 25, 26], we did not find a detectable increase in IFN-alpha or IFN-gamma proteins in NW, even at early time points after infection. This could be due to the low sensitivity of the assay used, a dilution effect due to the nasal wash procedure or evidence for active inhibition of interferon release by SARS-CoV-2 [37], as suggested by the relatively low concentration of interferons detected in serum of COVID-19 patients [38] or in vitro [39]. In addition, in silico analysis of the transcriptome led to the suggestion that macrophages, neutrophils, and activated dendritic cells are recruited in the nasopharyngeal epithelium of infected patients [23]. The fact that we detected more IL-8 and CCL2, as well as GM-CSF in NW support this finding but a more detailed analysis of the recruited cells in the compartment is needed, especially compared with the lungs [11, 40].

We observed no correlation between NW and serum cytokine concentrations. This discordance is well known for respiratory viral infections [15, 16] and underscores the likelihood that other sites such as the oral cavity also contribute to the overall host response [41]. It has the potential for misleading conclusions when assessments of SARS-CoV-2 immunopathology focus exclusively on the blood compartment, underestimating the role of the mucosal immune system especially early in infection. Serum cytokines therefore do not fully reflect viral responses at primary sites of infection.

In contrast to reports assessing single time points, this study provides a longitudinal analysis of the immune response in the upper respiratory tract in the course of mild disease, which represents the vast majority of cases worldwide. We show that local cytokine production is sustained in most patients until 2–3 weeks after disease onset, even when symptoms have subsided. Infectious particles are generally detected within the first week of infection, but viral RNA may be detected much later. Immune detection of viral RNA itself may thus be the main trigger of innate response in the epithelium, as previously shown for SARS-CoV [42]. Nonetheless, although our sample size remains limited, this sustained response does not lead to specific symptoms in the upper respiratory tract, but could rather be important in the induction of SARS-CoV-2-specific mucosal immunity during mild disease. We could not link the presence of high levels of CXCL10 or IL-6, indicative of a robust antiviral response, to loss of olfactory function, which may therefore rely on other mechanisms [43, 44].

The intensity of the local inflammatory response after mild SARS-CoV-2 infection seems overall less pronounced than that reported for other viral respiratory infections such as influenza [22, 29], respiratory syncytial virus, or rhinoviruses [45–47]. In addition to potential inhibition of the interferon pathway, an impairment of inflammasome/IL-1 beta induction as compared to other viruses was also a proposed mechanism [22]. In line with these results, we did not detect a significant change in IL-1Ra or IL-1-beta in NW. This relatively moderate antiviral response to SARS-CoV-2 may explain the high viral load in the upper respiratory tract and higher transmission rates.

We observed that only some patients had S1-specific IgA in the nasal cavity while almost none had IgG, the magnitude of the response was lower than the one measured in serum. These heterogeneity of the S1-specific IgA levels at the mucosa has been recently reported by others [48–50]. IgA is important for early neutralization of SARS-CoV-2 in blood and saliva [41]. Whether these specific local IgA plays a role in preventing severe disease early during infection requires further studies.

Our study had limitations. The size of the cohort is small, mainly due to the limited access to infected patients at the end of the first wave of COVID-19 in Geneva and practical constraints linked to performing the procedure during home visits at multiple time points. Patients had mild disease so our data should not be extrapolated to more severe disease. Enrolled participants were already RT-PCR positive at inclusion, at a median of 5 DPOS, so we could not capture early antiviral response during incubation time before symptoms onset. We could not reliably isolate RNA or phenotype the cells present in the NW samples due to the low number of cells recovered. The relatively low sensitivity of the assay used to measure cytokine and the dilution of the samples due to the nasal wash procedure may have limited our ability to detect cytokine production locally. Lack of correlation with symptoms should be interpreted with caution as symptoms were mainly self-reported and could therefore be subjective, although we believe that lack of correlation with anosmia remains solid despite limited sample size.

## Conclusion

High levels of hallmark cytokines associated with SARS-Cov2 infection such as CXCL10 and IL-6 could be detected in the nasal cavity of patients with mild COVID-19. The highest levels were detectable within the first week of symptoms and persisted for up to three weeks; they correlated with viral load but not with specific respiratory symptoms including anosmia. Mucosal S1-specific IgA could be detected in some patients.

## Supplementary Information

Below is the link to the electronic supplementary material.

Supplementary file1 (PDF 808 kb)

## Data Availability

The authors confirm that the de-identified data supporting the findings of this study are available within the article and supplementary data.
