# Supplementary material for: Longitudinal Analysis of Inflammatory Response to SARS-CoV-2 in the Upper Respiratory Tract Reveals an Association with Viral Load, Independent of Symptoms
Source: J Clin Immunol. 2021 Sep 28;41(8):1723–32. doi: 10.1007/s10875-021-01134-z (PMC8476983; doi:10.1007/s10875-021-01134-z)
Supplement: Supplementary file 1 — Supplementary file1 (PDF 808 kb) [file 10875_2021_1134_MOESM1_ESM.pdf]

# How immune responses shape virological and clinical characteristics of COVID-19: a prospective cohort study

**Short title: Understanding COVID-19**

| | |
|---|---|
| Research legislation: | Ordinance on human research with the exception of Clinical trials (HRO) [1]. |
| Type of Research Project: | Research project involving human participants |
| Risk Categorisation: | Category A |
| Project Leader: | Professor Claire-Anne Siegrist<br>Rue Michel-Servet 1<br>Geneva, Switzerland 1206<br>Tel. +41 79 213 9290<br>Claire-anne.siegrist@unige.ch |

**PROTOCOL SIGNATURE FORM**

| | |
|---|---|
| Study Title | **How immune responses shape virological and clinical characteristics of COVID-19: a prospective cohort study** |

The project leader has approved the protocol version 1.3, dated 7 May 2020, and confirms hereby to conduct the project according to the protocol, the Swiss legal requirements, current version of the World Medical Association Declaration of Helsinki and the principles and procedures for integrity in scientific research involving human beings.

**Project leader:**

Professor Claire-Anne Siegrist
Rue Michel-Servet 1
Geneva, Switzerland 1206
Tel. +41 79 213 9290
claire-anne.siegrist@unige.ch

Date: 7.05.2020______________________Signature: ________________________________

## GENERAL INFORMATION/ STUDY ADMINISTRATIVE STRUCTURE

| | |
|---|---|
| **Study type** | Prospective observational cohort study |
| **Study registration** | www.clinicaltrials.gov NCT04329546 |
| **Sponsor & Sponsor Representative** | Professor Claire-Anne Siegrist<br>University Hospitals of Geneva<br>Rue Gabrielle-Perret-Gentil 4<br>Geneva, Switzerland 1205 |
| **Study site** | University Hospitals of Geneva<br>Rue Gabrielle-Perret-Gentil 4<br>Geneva, Switzerland 1205 |
| **Central laboratories** | University Hospitals of Geneva<br>Rue Gabrielle-Perret-Gentil 4<br>Geneva, Switzerland 1205 |
| **Immunology laboratory** | Center for Vaccinology, University of Geneva |
| **Clinical study coordinator** | Doctor Angela Huttner, Center for Vaccinology, University of Geneva |
| **Lead clinical investigator** | Professor Laurent Kaiser, Infectious Diseases and National Reference Center for Emerging Viruses, HUG |
| **Lead virology evaluator** | Professor Isabella Eckerle, National Reference Center for Emerging Viruses, HUG |
| **Lead immunology evaluator** | Professor Claire-Anne Siegrist, Center for Vaccinology, University of Geneva |
| **Current protocol version** | 1.3 |

**Version history**

| Date | Version | Changes | By |
|---|---|---|---|
| 9.3.2020 | 1.0 | NA | CAS, AH, CSE, IE, BM, PV, GBR, AL, KP, AD |
| 20.3.2020 | 1.1 | Updating of epidemic statistics, removal of compensation for participants, adapted definition of cases, simplification of study plan, precisions on statistical analyses and ethics considerations. | CAS, AH |
| 17.04.2020 | 1.2 | Simplification of study schedule, precisions regarding informed-consent procedure; addition of two substudies | AH, AD, DVC, AL, CAS |
| 7.05.2020 | 1.3 | Addition of a final D28 for negative contacts; addition of nasal wash as a sampling option for children and contacts. | AH, CAS |

## TABLE OF CONTENTS

GENERAL INFORMATION/ STUDY ADMINISTRATIVE STRUCTURE 3
TABLE OF CONTENTS 4
GLOSSARY OF ABBREVATIONS 7
PROTOCOL SYNOPSIS 8
1 BACKGROUND AND PROJECT RATIONALE 11
1.1 Background 11
1.2 Rationale 11
2 PROJECT OBJECTIVES AND DESIGN 11
2.1 Hypothesis and primary and secondary objectives 11
2.2 Primary and secondary endpoints 12
2.2.1 Primary outcome measures 12
2.2.2 Secondary outcome measures 13
2.3 Project design 14
3 PROJECT POPULATION AND STUDY PROCEDURES 14
3.1 Project population, inclusion and exclusion criteria 14
3.1.1 Inclusion criteria 14
3.1.2 Exclusion criteria 15
3.2 Recruitment, screening and informed consent procedure 15
3.3 Study procedures 16
3.3.1 Study visits for adult case patients 16
Schedule of assessments for adult patients 16
*Visit 1, Enrollment (Day 1)* 17
*Day 3 (±1) after diagnosis/suspected diagnosis* 19
*Visit 2 (Day 7 [±2])* 19
*Day 10 (±1) after diagnosis/suspected diagnosis* 19
*Visit 3 (Day 14 [±3]) after diagnosis/suspected diagnosis* 19
*Visit 4 (Day 28 [±7]) after diagnosis/suspected diagnosis* 20
*Visit 5 (Day 56 [±14]) after diagnosis/suspected diagnosis* 20
3.3.2 Study visits for pediatric case patients 20
Schedule of assessments for pediatric case patients 20
3.3.3 Study visits for adult household contacts 22
*Visit 1, Enrollment (Day 1)* 23
*Visit 2 (Day 3 [±1])* 23
*Visit 3 (Day 7 [±2])* 24
*Visit 4 (Day 10 [±2])* 24
*Visit 5 (Day 14 [±3])* 24
*Visit 6 (Day 28 [+14])* 24
3.3.4 Study visits for pediatric household contacts 24

3.4 Withdrawal and discontinuation 25
4 STATISTICS, METHODOLOGY AND LABORATORY ANALYSES 26
4.1. Statistical analysis plan 26
4.1.1 Sample size 26
4.1.2 Statistical analysis plan 26
4.1.3 Specific immunological analyses 27
4.1.4 Specific virologic analyses 27
4.2. Handling of missing data 28
4.3. Potential biases and risk mitigation strategies 28
5 OBSERVATIONAL SUBSTUDIES 29
5.1 Nested prospective observational cohort study on cytokine responses and viral load in nasal wash 29
5.1.1 Background and rationale 29
5.1.2 Nested nasal-wash study design, setting and population 29
5.1.3 Nested nasal-wash study outcome 30
5.1.4 Nested nasal-wash study statistical considerations 30
5.1.5 Nested study structure 30
5. 2 Substudy: PERSISTENCE of humoral and cellular immunity against SARS-CoV-2 30
5.2.1 Background and rationale for the PERSISTENCE substudy 30
5.2.2 The PERSISTENCE substudy design, setting and population 30
5.2.3 Study outcomes of the PERSISTENCE substudy 31
5.2.4 Laboratoy methods for the PERSISTENCE substudy 32
5.2.5 Statistical considerations for the PERSISTENCE substudy 32
5.2.6 Ethical considerations for the PERSISTENCE substudy 32
5.2.7 Data handling for PERSISTENCE substudy 32
5.2.8 Substudy structure 32
6 REGULATORY ASPECTS AND SAFETY 33
6.1 Local regulations / Declaration of Helsinki 33
6.2 Notification of safety and protective measures (HRO Art. 20) 33
6.3 Serious events (HRO Art. 21) 33
6.4 Biosafety procedures 33
6.4.1 Personnel safety during study visits 33
6.4.2 Laboratory biosafety 33
6.5 Amendments 34
6.6 End of project 34
6.7 Insurance 34
7 FURTHER ASPECTS 34
7.1 Overall ethical considerations 34
7.2 Risk-Benefit Assessment 35
7.3 Rationale for the inclusion of vulnerable participants 35
8 QUALITY CONTROL AND DATA PROTECTION 36

8.1 Quality measures 36
8.2 Data recording and source data 36
8.3 Confidentiality and coding 37
8.4 Retention and destruction of study data and biological material 37
9 FUNDING / PUBLICATION / DECLARATION OF INTEREST 38
10 REFERENCES 38

## GLOSSARY OF ABBREVATIONS

| | |
|---|---|
| AE | Adverse event |
| COVID-19 | Coronavirus Disease (2019) |
| CRF | Case report form |
| EC | Ethics committee |
| ELISA | Enzyme-linked immunosorbent assay |
| ELISPOT | Enzyme-linked immunospot |
| GMT | Geometric mean titer |
| HRA | Human Research Act |
| HUG | University Hospitals of Geneva (Hôpitaux universitaires de Genève) |
| ICF | Informed consent form |
| ITT | Intention to treat |
| LPTh | Loi sur les produits thérapeutiques (Therapeutic products law) |
| MNT | Microneutralization test |
| LRH | Loi relative à la recherche sur l'être humain (Law on Human Subjects Research) |
| PBMC | Peripheral blood mononuclear cells |
| PII | Personally identifiable information |
| pfu | Plaque-forming unit |
| PP | Per protocol |
| PRNT | Plaque reduction neutralization test |
| rANOVA | Repeated measure analysis of variance |
| rIFA | Radio-immunofocus assay |
| SARS-CoV-2 | Severe-acute-respiratory-syndrome-coronavirus-2 |
| WHO | World Health Organization |

## PROTOCOL SYNOPSIS

| PROTOCOL SYNOPSIS – UNDERSTANDING COVID-19 | |
|---|---|
| PROTOCOL TITLE: | How immune responses shape virological and clinical characteristics of COVID-19: a prospective cohort study |
| PRINCIPAL INVESTIGATOR: | Prof. Claire-Anne Siegrist, MD |
| STUDY CENTER: | University Hospitals of Geneva, Geneva, Switzerland 1205 |
| SPONSORING INSTITUTION: | University Hospitals of Geneva<br>Rue Gabrielle-Perret-Gentil 4, Geneva, Switzerland 1205 |
| CLINICAL STUDY COORDINATOR: | Dr. Angela Huttner, MD |
| CLINICAL INVESTIGATORS | Dr. Diego Andrey, MD PhD, Dr. Géraldine Blanchard, MD, Dr. Christiane Eberhardt, MD, Dr. Angela Huttner, MD, Prof. Laurent Kaiser, MD, Dr. Arnaud L'Huillier, MD, Dr. Natasha Loevy, MD, Prof. Klara Posfay-Barbe, MD, Dr. Pauline Vetter, MD, Dr. Diem-Lan Vu Cantero, MD, PhD |
| VIROLOGY EVALUATORS: | Dr. Pascal Cherpillod, PhD, Prof. Isabella Eckerle, MD, Prof. Laurent Kaiser, MD, Dr. Manuel Schibler, MD, Prof. Caroline Tapparel, PhD, Dr. Pauline Vetter, MD, Dr. Diem-Lan Vu Cantero, MD, PhD |
| IMMUNOLOGICAL EVALUATORS: | Dr. Géraldine Blanchard, MD, PhD, Prof. Arnaud Didierlaurent, PhD, Dr. Christiane Eberhardt, MD, Dr. Benjamin Meyer, PhD, Prof. Claire-Anne Siegrist, MD, Dr. Maria Vono, PhD |
| STUDY DESIGN: | Single-center prospective observational cohort study |
| STUDY POPULATION: | Adults and children with laboratory-confirmed or strongly suspected SARS-CoV-2 infection and their household contacts |
| SAMPLE SIZE: | N=50 infected patients, plus their household contacts, for a maximum of 250 participants; (Persistence substudy: N=200 infected hospital workers) |
| FOLLOW-UP PERIOD: | Patients: 8 weeks; Household contacts: 2 weeks; Persistence substudy: 2 years |
| STUDY PERIOD: | March 2020 – March 2022 |
| RATIONALE: | The novel coronavirus, named 'severe acute respiratory syndrome coronavirus 2 (SARS-CoV-2) is spreading worldwide, yet requirements (viral load, proximity, symptom severity) for effective transmission are still unknown. While the epidemic began in China, countries in the Middle East, Africa, and Europe are experiencing exponential increases in their numbers of clinical COVID-19 cases. Several European countries and much of the US have essentially been placed on lockdown, as governments move from all-out containment efforts to intensive mitigation.<br><br>No proven effective treatment or vaccine exists. Initial efforts have focused on slowing viral propagation through hygiene measures, isolation of cases and social distancing – and are now moving towards the protection of the most vulnerable. As a growing number of patients requiring hospitalization for medical care is soon expected in Switzerland, a change of strategy will likely include home quarantine of confirmed cases and their household contacts – with only those in need of medical care being hospitalized.<br><br>The virus and its activity, clinical manifestations, and transmissibility in humans are poorly understood. **The hypothesis we will assess is that symptoms (or lack thereof) are primarily driven by the magnitude of specific and/or cross-reactive immune responses, at baseline or in response to SARS-CoV-2 infection, which could explain** |

| | |
|---|---|
| | **both the benign nature of pediatric disease and the severity of diseases in elderly and/or immunosuppressed patients.** |
| PRIMARY OBJECTIVE: | The primary aim of this prospective observational study is to establish how immune responses to SARS-CoV-2 or to cross-reactive viral pathogens correlate with the virological and clinical characteristics of SARS-CoV-2-infected patients and their exposed household contacts. |
| SECONDARY OBJECTIVES: | Secondary objectives are:<br>- To characterize the daily evolution and severity of clinical symptoms in previously healthy or vulnerable SARS-CoV-2-infected patients.<br>- To characterize viral kinetics over time (viral load and shedding [location, duration, etc.])<br>- To characterize the innate and adaptive immune response to SARS-CoV-2 infection, at baseline, until symptom resolution and during convalescence.<br>- To identify potential biomarkers of disease progression or remission. |
| EXPLORATORY OBJECTIVES: | Exploratory objectives are:<br>- To assess the effect of pre-existing conditions (age, co-morbidities, medication, habits [smoking, etc.]) on disease severity, viral load and immune parameters<br>- To assess the existence of mutations in clinical isolates of SARS-CoV-2 and their association with the severity of the symptoms and/or virological or immune parameters<br>- The presence and quantity of viral RNA in body fluids (shedding) at baseline and at follow-up time points, as quantified by RT-PCR<br>- Virus isolation and preparation of viral stocks for future *in vitro* experiments<br>- To analyze pre-existing immunity to circulating human pathogenic CoVs (HCoV-229E, -NL63, -OC43 and –HKU1) and other viral pathogens, including existing antibody, T-cell and memory B-cell immunity. Results will be correlated with disease severity during SARS-CoV-2 infection, following the hypothesis that severe clinical symptoms may result from the reactivation of pre-existing cross-reactive memory T or B cells (which may be more abundant in senior citizens).<br>- To analyze the antibody repertoire and functionalities of antibody specific to the Spike (S) of SARS-Cov-2 and association with disease severity.<br>- To compare the local (sputum, BAL, saliva) and systemic (blood) cytokine and cellular response during infection and to assess associations between HLA-phenotype and disease severity.<br>- To identify the molecular and cellular determinants of the severity of disease. This will be approached by analyzing potential correlations with markers of innate responses elicited during the first days after infection, T cell and B cell responses at various time points and onset/magnitude of antibody titers – and disease severity. |
| PROCEDURE AND FOLLOW-UP: | **Patients with confirmed infection** (and/or high suspicion/actively being treated as CoVID+ cases pending test confirmation) will be identified by means of the hospital's surveillance network, which includes laboratory notification of specimen positive for SARS-CoV-2 testing. The temporary inclusion of "highly suspect cases", e.g. contacts of known COVID-19 patients, is now necessary because of the delay between testing and results.<br><br>As of April 17th, 2020, testing focuses on patients with active disease and healthcare workers (regardless of disease severity).<br><br>Those granting informed consent will undergo 1) evaluation and sample collection every 48-72h if/while hospitalized, 2) five study visits over eight weeks with collection of |

| | |
|---|---|
| | biological samples at these visits and at interval/prespecified time points (see study schedule):<br>- Non-invasive samples from various body fluids (and bronchoalveolar lavage [BAL] specimen if performed for clinical reasons) will be collected for detection and quantification of viral RNA and infectious virus, and for measurements of mucosal immune responses<br>- Blood will be collected for characterization of infection-associated kinetics of relevant markers and innate/adaptive immune responses<br><br>**Household contacts** will be contacted at time of diagnosis/suspected diagnosis of the index case.<br>- After granting informed consent, they will undergo a baseline evaluation followed by repeat evaluation over 14 days (see below).<br>- If their PCR becomes positive at any point, they will be followed-up as infected patients.<br>- If PCR remains negative, they may be invited for final antibody sampling on day 28.<br>*Nb:* Blood collection will be adapted to body weight in pediatric patients (see below). |
| PARTICIPANT INCLUSION CRITERIA: | - Patients of all ages with laboratory-confirmed SARS-CoV-2 infection (and/or high suspicion/actively being treated as CoVID+ cases pending test confirmation), regardless of the presence/absence or severity of symptoms<br>- Household contacts of patients with laboratory-confirmed SARS-CoV-2 infection (and/or high suspicion/actively being treated as CoVID+ cases pending test confirmation) |
| PARTICIPANT EXCLUSION CRITERIA: | - Unable to provide written or oral-witnessed informed consent AND without patient representative able to provide written or oral-witnessed informed consent |
| STATISTICAL ANALYSIS: | The sample size will not be calculated; it will be determined by the number of patients diagnosed and willing to participate, and by the level of funding study investigators can secure. At the time of submission, at least 50 patients plus their household contacts are expected to be included, for a maximum of 250 participants.<br>Descriptive statistics will be employed. Logistic and linear regression models will be constructed to evaluate associations among viral load (plasma and respiratory samples), the kinetics of viral load (i.e., slope of ascent or descent), symptom burden, antibody titers, and overall clinical outcome. Where appropriate, highly skewed data will be log-transformed and presented as geometric means with 95% confidence intervals. Exploratory multiparametric tests including dimension reduction, distances matrices and supervised clustering, may also be applied. Associations with P values of ≤.05 (two-sided) will be considered statistically significant. |
| STUDY DURATION: | 2 years (expected) |

# 1 BACKGROUND AND PROJECT RATIONALE

## 1.1 Background

The novel coronavirus, named 'severe acute respiratory syndrome coronavirus 2 (SARS-CoV-2)', was first detected in China in December 2019.[1] Its clinical manifestations in humans appear to be wide ranging, from asymptomatic to lethal infections, while its human-to-human transmission remains poorly understood. To date, the majority of the >100,000 reported infections are concentrated in China.[2] Yet Middle Eastern, African, and European countries are experiencing exponential increases in the number of clinical COVID-19 cases. Several European countries and much of the US have essentially been placed on lockdown, as governments move from all-out containment efforts to intensive mitigation. No proven treatment or vaccine exists, though efforts for both are underway.

## 1.2 Rationale

The aims of this prospective observational study are to characterize the viral properties of SARS-CoV-2 (infection and shedding kinetics), human host responses (clinical manifestations, innate and humoral immune responses), identifying clinical and immunological risk factors for or immune markers predictive of higher symptom burden and increased/prolonged shedding. Survivor serum will be collected for the characterization of anti-SARS-CoV-2 B cell responses and antibodies in a larger effort to construct monoclonal antibody (mAB) therapies.

# 2 PROJECT OBJECTIVES AND DESIGN

## 2.1 Hypothesis and primary and secondary objectives

The hypothesis we will assess is that symptoms (or lack thereof) are primarily driven by the magnitude of specific and/or cross-reactive immune responses, at baseline or in response to SARS-CoV-2 infection, which could explain both the benign nature of pediatric disease and the severity of diseases in elderly and/or immunosuppressed patients.

The primary aim of this prospective observational study is to establish how immune responses correlate with virological and clinical characteristics of SARS-CoV-2-infected patients and exposed household contacts (defined as those sleeping in the same apartment/house as the infected patients).

Secondary objectives are:

- To characterize **the daily evolution and severity of clinical symptoms** in previously healthy or vulnerable (co-morbidity, medication, etc.) SARS-CoV-2-infected patients.
- To characterize **viral kinetics** in infected human hosts (viral load and shedding [form, duration, and association with symptom burden])
- To characterize **the innate and adaptive immune responses** to SARS-CoV-2 infection:
    - kinetics, type and magnitude of innate response to infection
    - kinetics and quality of B- and T-cell responses to infection
- To identify **potential biomarkers of disease progression or remission**.

Exploratory objectives will include:

**Clinical symptoms and scores:**

- To assess the effect of pre-existing conditions (age, co-morbidities, medication, habits [smoking, etc.]) on disease severity, viral load and immune parameters

**Virology:**

- To assess the existence of mutations in clinical isolates of SARS-CoV-2 and their association with the severity of the symptoms and/or virological or immune parameters
- The presence and quantity of viral RNA in body fluids (shedding) at baseline and at follow-up time points, as quantified by RT-PCR
- Virus isolation and preparation of viral stocks for future *in vitro* experiments

**Immunology:**

- To analyze pre-existing immunity to circulating human pathogenic CoVs (HCoV-229E, -NL63, -OC43 and –HKU1) and other viral pathogens, including existing antibody, T-cell and memory B-cell immunity. Results will be correlated with disease severity during SARS-CoV-2 infection, following the hypothesis that severe clinical symptoms may result from the reactivation of pre-existing cross-reactive memory T or B cells (which may be more abundant in senior citizens).
- To analyze the antibody repertoire and functionalities of antibody specific to the Spike (S) of SARS-CoV-2 and association with disease severity.
- To compare the local (sputum, BAL, saliva) and systemic (blood) cytokine and cellular response during infection and to assess associations between HLA-phenotype and disease severity.

**All:**

- To identify the molecular and cellular determinants of the severity of disease. This will be approached by analyzing potential correlations with markers of innate responses elicited during the first days after infection, T cell and B cell responses at various time points and onset/magnitude of antibody titers – and disease severity.

Note: we have selected to follow patients until day 56 as 1) symptoms may persist beyond one month in severe cases, 2) virus may be detected later than one month in some cases, at least as RNA particles, and 3) certain immune responses (such as the induction of memory responses) require at least 6 weeks to mature.

## 2.2 Primary and secondary endpoints

### <u>2.2.1 Primary outcome measures</u>

**The co-primary immunogenicity outcome measure** is the geometric mean antibody concentration of total IgG antibodies to SARS-CoV-2 assessed by ELISA at 28 days after diagnosis/suspected diagnosis.

**The co-primary virology outcome measure** is the peak viral load in the 56 days following diagnosis/suspected diagnosis, quantified by real-time reverse-transcriptase polymerase chain reaction (RT-PCR) on nasopharyngeal swabs.

**The co-primary clinical outcome of disease** is the maximal symptom severity in the 56 days following diagnosis/suspected diagnosis, assessed by the WHO-defined categories for SARS-COV-2 induced disease as:

- <u>asymptomatic</u>
- <u>mild</u> (no pneumonia)

- moderate (pneumonia – clinical + X-ray or CT scan)
- severe (dyspnea, tachypnea ≥ 30/min, blood oxygen saturation ≤ 93%, PaOW/FiO2 ratio < 300 and/or lung infiltrates > 50% of the lung field within 24-48h)
- critical (respiratory failure, septic shock and/or multiple organ dysfunction/failure)

Each patient will be ascribed to one of these categories by the clinical investigator. To account for the yet unknowns, other criteria than those above (example: cardiac dysfunction) may have to be used for severity categorization. This will occur at the sole discretion of the clinical investigator.

2.2.2 Secondary outcome measures

If not indicated here, the time points of all outcome measurements can be found in the Schedule of Assessments and in the descriptions of individual visits (Chapter 4).

**Clinical symptoms and scores:**

- Clinical symptoms including fever, cough, fatigue, shortness of breath, respiratory rate, sore throat, headache, myalgia or arthralgia, chills, nausea or vomiting, nasal congestion or rhinorrhea, diarrhea, hemoptysis, conjunctivitis, skin rash/lesions, *etc*.
- Any other relevant symptom according to the evolution of our knowledge of SARS-CoV-2 infection
- Baseline general severity score (National Early Warning Score [NEWS] 2,[3] quick Sepsis Organ-related Failure Assessment [qSOFA][4]), underlying conditions (Charlson comorbidity index[5]), treatments at diagnosis/suspected diagnosis (all), habits (smoking, BMI)
- Pneumonia severity scores (CURB,[6] FINE,[7] others as deemed useful by the clinical investigator)
- Grading of the severity of imaging data (chest X-ray or CT scan): single/multilobar, uni/bilateral, type of lesions, progression over time

**Virology**:

- The presence and quantity of viral RNA in plasma at baseline and at follow-up time points, as quantified by RT-PCR
- The presence and quantity of infectious viral particles ("viable virus") in specimens described above, at time points as above, by inoculation of participant samples in cell lines in the BSL-3 laboratory
- Viral isolates will be kept for further *in vitro* experiments (co-infections with respiratory viruses or respiratory bacteria, infections in presence or absence of antibodies developed in response to infections by other coronaviruses, antiviral testing)

**Immunology:**

- Cytokines or other inflammatory markers present in the plasma at various time points
- Changes in innate or inflammatory pathways identified by gene expression in whole-blood
- Phenotype and activation levels of the main innate cell subsets in blood (including but not limited to neutrophils, monocytes, dendritic cells, natural killer cells…)

- Magnitude and quality (antibody avidity, glycosylation profile) of SARS-CoV-2-specific serum IgG, IgM and IgA antibody titers on days 0 (baseline), 7 and 14 (acute phase), 28 and 56 (convalescent phase) after diagnosis/suspected diagnosis
- SARS-CoV-2 -specific sputum IgG and IgA antibody titers to assess quality and quantity of the mucosal humoral immune response
- Neutralizing capacity of serum and sputum antibodies will be assessed at the indicated times by either plaque reduction- (PRNT) or microneutralization tests (MNT)
- Kinetics and quality of cellular adaptive immune responses will be measured in isolated PBMCs during the phase of infection on days 0, 7, 14 and 28 after symptom onset,
    - Spike (S) protein-specific circulating antibody-secreting B cells and memory B cells will be assessed by ELISpot
    - The magnitude, activation status and phenotype of B cells, CD4 and CD8 T cells will be assessed by flow cytometry and RNA sequencing
    - Functionality and cytokine profile of antigen-specific CD4 and CD8 T cells will be quantified by ELISpot and flow cytometry
    - In a subset of high-responders, monoclonal antibodies will be generated from individually sorted antigen-specific antibody-secreting cells

In a subset of patients who will undergo bronchoalveolar lavage, lung biopsies or autopsy, lung tissue-resident T cell memory responses will be quantified and compared to the memory T cells responses detectable in the peripheral blood (ELISpot; flow cytometry, RNA sequencing). Immunostaining for viral antigens will be performed on biopsies or autopsy tissue sections to highlight the infected cell type and the inflammation level.

### 2.3 Project design

This a single-center prospective observational cohort study.

## 3 PROJECT POPULATION AND STUDY PROCEDURES

### 3.1 Project population, inclusion and exclusion criteria

#### 3.1.1 Inclusion criteria

The following persons may be included:

- A patient of any age meeting the European Centre for Disease Control and Prevention's confirmed case definition:[2] "A person with laboratory confirmation of virus causing COVID-19, irrespective of clinical signs and symptoms" (and/or high suspicion/actively being treated as CoVID+ cases pending test confirmation)
- Household contacts (defined as those sleeping in the same apartment/house as an infected patient) of a patient with laboratory-confirmed COVID-19, whether symptomatic or not, and whether testing positive or not during a period of 14 days

This will allow for the inclusion of patients requiring hospital admission (*i.e.*, with moderate, severe or critical symptoms) as well as patients likely to develop no or mild symptoms (health care workers [who will continued to be screened at the HUG] and household contacts who will eventually become infected).

It will also allow for the inclusion of patients with a high suspicion/actively being treated as CoVID+ cases pending test confirmation. Indeed, insufficient resources compared to the needs are slowing down the test procedure and increasing the intervals until confirmation. The study may be terminated early for participants whose diagnosis could not be laboratory-confirmed and whose symptoms ultimately were attributed by the attending physician to an alternate diagnosis.

#### 3.1.2 Exclusion criteria

The only exclusion criterion is long-term incapacity leading to the inability to provide written or oral-witnessed informed consent WHILE not having a patient representative with the ability to provide written informed consent. In the case of patients with what is clinically judged to be *transient* incapacity due to acute coronavirus infection (hypoxia, intubation and sedation, etc.), the patient may be included for data and sample collection (as described below in Section 6.3); *these data and samples, however, will not be used until the patient recovers capacity and provides written or oral-witnessed informed consent*.

Patients with ongoing signs of infection at the recovery/convalescent time points, *i.e.*, potentially infected by other viruses, will be assessed in subgroup analyses.

Enrollment in a separate clinical trial will not preclude participation in this observational study; patients given experimental therapies will be assessed in subgroup analyses.

Pregnancy is not an exclusion criterion.

### 3.2 Recruitment, screening and informed consent procedure

Recruitment will take place at HUG or at home. Patients with confirmed infection will be identified by means of the hospital's surveillance network, which includes laboratory notification of specimen positive for SARS-CoV-2 testing. If not hospitalized or present at time of diagnosis/suspected diagnosis, they and their household contacts will initially be contacted by telephone.

The study information and informed consent form (ICF) will be made available to patients/contacts in advance via a phone contact followed by an electronic message containing a PDF of the study information brochure when possible. This will give the patient/contacts sufficient time and opportunity to inquire about details of the study and to decide whether or not to participate in the study before the first visit. Consenting participants must sign and date the ICF before any study-specific procedures may be performed. At the first study visit, the participant will be fully informed of all aspects of the study, the potential risks and their obligations. The following general principles will be emphasized:

- Participation in the study is entirely voluntary.
- Refusal to participate involves no penalty or loss of medical benefits.
- The participant may withdraw from the study at any time, without supplying a reason for withdrawal.
- The participant is free to ask questions at any time to allow him or her to understand the purpose of the study and the procedures involved.
- There is no direct benefit from participating.

The aims of the study and all sampling and blood draws to be carried out will be explained. If the patient/contact or his/her representative does provide consent to participate, he or she, or his/her patient representative, will sign and date two copies of the consent form, one for his/her personal records, and one to be stored at site in the source documents. In the rare instance that a patient/contact

has cognitive capacity but is physically unable to provide a written signature, oral consent will be accepted, per Article 8 of the Swiss Ordinance on Clinical Trials in Human Research (810.305), as long as an independent witness (an individual who is not part of the study team) can provide written testimony that the patient received the information and provided oral consent. These forms will also be signed and dated by the Investigator.

### 3.3 Study procedures

All patients and household contacts will be asked to remain in the study throughout its entire duration. If a participant (who is not/no longer hospitalized) does not appear at a scheduled visit, every effort will be made to contact him or her to reschedule the visit.

**As patients not requiring medical care are now required to self-quarantine at home, home visits will be organized.**

The study visits are described in detail below. An overview is found in Tables 1-4 (Schedules of Assessments).

Each visit is assigned a time point and a window period within which the visit will be conducted. Deviations from the window periods are discouraged, but are permitted at the discretion of the Investigator in the interest of completing the study schedule and obtaining clinical and immunological data.

Data (clinical history) will be recorded by the collaborators of the study using the specific CRF.

Coded samples will be stored in the virology laboratory at -80°C (liquid nitrogen for cells) while awaiting further assessment.

3.3.1 Study visits for adult case patients

Schedule of assessments for adult patients

Five visits will occur for all included patients, with additional specimen collection for hospitalized patients. Day 0 is the day of the diagnostic test and day 1 is the day of inclusion.

Table 1. Schedule of assessments for adult case patients.

| **Visit** | **Routine** | **Inclusion** | **Follow-up** | | | | | |
|---|---|---|---|---|---|---|---|---|
| **Visit number** | | **1** | **1.1*** | **2** | **2.1*** | **3** | **4** | **5** |
| **Timeline (day 0 = day of diagnostic test)** | | 1 | 3 | 7 | 10 | 14 | 28 | 56 |
| **Window period (days)** | | -1,+2 | ±1 | ±2 | ±1 | ±3 | ±7 | ±14 |
| Informed consent | | X | | | | | | |
| Entry criteria | | X | | | | | | |
| Detailed clinical and exposure history | | X | | | | | | |
| Physical exam | X | X | | (X) | | (X) | (X) | (X) |
| Clinical symptoms severity evaluation | | X | | X | | X | X | X |
| Chest imaging (X-ray ± CT) | (X) | | | (X) | | (X) | (X) | |
| Nasopharyngeal swab** | X | | X | X | X | X | X | X |
| Oropharyngeal swab** | | X | X | X | X | X | X | |
| Nasal wash | | (X) | (X) | (X) | (X) | (X) | | |
| Bronchoalveolar lavage specimen collection** | (X) | | (X) | (X) | (X) | (X) | (X) | |
| Sputum specimen collection*** | | X | X | X | X | X | X | X |

| | | | | | | | | |
|---|---|---|---|---|---|---|---|---|
| Other body fluid (tears, vesicles, stools…) if clinically appropriate | (X) | | (X) | (X) | (X) | (X) | (X) | |
| Complete blood count and cell distribution | 2 | 2 | 2 | 2 | 2 | 2 | 2 | 2 |
| Blood chemistry (NaCl, BUN, creatinine, AST, ALT, alkaline phosphatase, CRP) ** | 2 | 2 | | (2) | | (2) | (2) | |
| HLA phenotyping for T cell responses | | 2 | | | | | | |
| Plasma for viral load and innate responses | | 2 | 2 | 2 | 2 | 2 | 2 | 2 |
| Blood for Innate cell phenotyping | | 2 | 2 | 2 | 2 | 2 | 2 | 2 |
| Blood RNA preservation | | 2 | 2 | 2 | | 2 | 2 | 2 |
| Serum collection (antibody assessment) | | 6 | 6 | 6 | | 6 | 6 | 6 |
| Cellular immune responses (PBMCs) | | 32 | | 32 | | 32 | 32 | 32 |
| Maximum blood volume (mL) | | 50 | 14 | 46 | 6 | 46 | 46 | 46 |
| Maximum cumulative blood volume (mL) | | 50 | 64 | 110 | 116 | 162 | 208 | **254** |

*Specimen collection will occur on D3 and D10 after diagnosis/suspected diagnosis only for patients who are still hospitalized.
**Repeat specimen collection will occur only if prior samples have not already normalized.
(X) Data will be collected / samples assessed only if these are performed for clinical reasons. Nasal wash may be offered with/as an alternative to nasopharyngeal swabbing in participants who may otherwise refuse the latter.

*** If productive cough only, for viral load, innate cytokines and detection of IgA & IgG antibodies.

*Visit 1, Enrollment (Day 1)*

This visit will occur on day 1, with day 0 being the day of diagnosis / suspected diagnosis. The following activities will occur during the first visit:

- The informed consent form (ICF) will be provided as described above; no further activity will take place until signing of the ICF
- Inclusion and exclusion criteria will be evaluated
- A detailed clinical and exposure (household, known contact, nosocomial, other, unknown) history will be taken and clinical severity evaluated.
- A full / targeted physical examination will be performed depending upon the presenting symptoms
- If chest imaging (X-ray or CT scan) is performed as part of the clinical work-up, its conclusions will be collected
- <u>Non-blood specimen collection</u>:
    - The nasopharyngeal swab will have been collected for diagnosis before inclusion; data will be recorded.
    - Oropharyngeal swab will be performed.
    - Nasal wash will be offered to 15 participants (see chapter 5 below) and may additionally be offered with/as an alternative to nasopharyngeal swabbing in participants who may otherwise refuse the latter.

- Bronchoalveolar lavage (BAL) specimens will only be collected / assessed if this procedure has been clinically indicated / performed.
- Stool/anal swab, sputum and/or other body fluids (tears, vesicles) specimens will only be harvested if clinically indicated (no sampling will be initiated for the sake of the study).

All available samples will be subjected to the following analyses:

- Assessment of viral load (RNA)
  - Association of viral load / shedding with clinical course/outcome and secondary cases in the household
- Other exploratory immunological measurements:
  - Assessment of the presence and quality of IgG and IgA antibodies by ELISA
  - Assessment of cytokines and cell phenotyping by flow cytometry
  - Kinetics and quality of antigen-specific antibody response (IgG and IgA) of sub-classes (IgG1-4) and of antibody avidity by ELISA
  - Persistence of antibodies during and after resolution of symptoms
  - Functional antibodies (neutralization assays (PRNT and/or MNT))
  - Characterization of tissue-resident T cells by flowcytometry and RNA sequencing (phenotype) and ELISpot (antigen-specificity and functionality) and comparing with responses detected in the blood

- <u>Blood specimen collection</u>:
  A maximum of 50 ml of blood (adults, adjusted to body weight for children) will be collected for:
  - Complete blood count and distribution, and main chemistry (electrolytes, renal and liver function)
  - Routine inflammatory marker (CRP)
  - HLA phenotyping for further assessment of T cell responses
  - Plasma viral load quantification
  - Assessment of innate response
    - Cytokines in serum measured by a multiplex assay
    - Viral mRNA levels measured by RNA seq and analysis of upregulated or down-regulated immune pathways
    - Phenotype of the main innate cells subsets in blood (including but not limited to neutrophils, monocytes, DC, NK cells..) by flow cytometry as well as expression of activation markers
  - Assessment of humoral adaptive response:
    - Assessment of the presence and quality of the humoral immune response by ELISA, rIFA, PRNT and/or MNT, isotypes (IgG, IgM and IgA) as well as subclasses ($IgG_{1-4}$) and antibody avidity by ELISA and rIFA
    - Antibody neutralization assays (PRNT and/or MNT)
  - Assessment of cellular adapted immune responses (from fresh or cryopreserved isolated PBMCs):

- Quantification of antigen-specific circulating antibody-secreting B cells during the acute phase by ELISpot and to assess the kinetics of the response
- Characterization of the magnitude, activation status and phenotype of B cells, CD4 and CD8 T cells by flow cytometry and in a subset of patients by RNA sequencing
- Assessing the functionality and the cytokine profile of antigen-specific CD4 and CD8 T cells after *in vitro* stimulation (ELISpot and flow cytometry)
- Assessing the kinetics of memory B cell development during infection and assessing their persistence in convalescent samples
- A subset of patients will be identified with a high frequency of SARS-CoV-2-specific antibody secreting cells. These cells will be isolated, their antibody-encoding region sequenced, and monoclonal antibodies will be generated *in vitro*. The monoclonal antibodies will further be tested to assess their specificity to SARS-CoV-2, their avidity and their neutralization capacity, thus showing their potential for later therapeutic use.

- Freezing and storage of serum for characterization of anti-SARS-CoV-2 antibodies in a later effort to construct monoclonal antibody (mAB) therapies

*Day 3 (±1) after diagnosis/suspected diagnosis*

Formal research visits will not occur on this day, though specimen collection will for patients who are still hospitalized: non-blood specimens identified in Table 1 will be collected for the analyses described under Visit 1 above and blood will be collected (see Schedule of Assessments).

*Visit 2 (Day 7 [±2])*

The following will occur/be collected:

- Targeted physical exam if the patient is still symptomatic/if clinically indicated
- Clinical symptoms severity evaluation
- Evolution of chest imaging if clinically appropriate
- All non-blood specimens as described above under Visit 1
- Harvesting of blood for the analyses identified in Table 1 and described under Visit 1

*Day 10 (±1) after diagnosis/suspected diagnosis*

Formal research visits will not occur on this day, though specimen collection will for patients who are still hospitalized: non-blood specimens identified in Table 1 will be collected for the analyses described under Visit 1 above and blood will be collected (see Schedule of Assessments).

*Visit 3 (Day 14 [±3]) after diagnosis/suspected diagnosis*

The following will occur/be collected:

- Targeted physical exam if the patient is still symptomatic/if clinically indicated
- Clinical symptoms severity evaluation
- Evolution of chest imaging if clinically appropriate
- All non-blood specimens as described above under Visit 1

- Harvesting of blood for the analyses identified in Table 1 and described under Visit 1

*Visit 4 (Day 28 [±7]) after diagnosis/suspected diagnosis*

The following will occur/be collected:

- Targeted physical exam if the patient is still symptomatic/if clinically indicated
- Clinical symptoms severity evaluation
- All non-blood specimens as described above under Visit 1
- Harvesting of blood for the analyses identified in Table 1 and described under Visit 1

*Visit 5 (Day 56 [±14]) after diagnosis/suspected diagnosis*

The following will occur/be collected:

- Targeted physical exam if the patient is still symptomatic/if clinically indicated and/or if previous findings have not already normalized
- Clinical symptoms severity evaluation
- Evolution of chest imaging if clinically appropriate
- All non-blood specimens as described above under Visit 1
- Harvesting of blood for the analyses identified in Table 1 and described under Visit 1

### 3.3.2 Study visits for pediatric case patients

Visits for pediatric case patients will follow those of adult case patients, but specimen collection will differ: no oropharyngeal specimens will be collected, and PBMCs will only be collected if the maximum blood volume would not exceed what is appropriate for body weight (see Table 2). See page 13 for methods to maximize small sampling for children.

#### Schedule of assessments for pediatric case patients

Five visits will occur for all included patients, with additional specimen collection for hospitalized patients. Day 0 is the day of diagnosis/suspected diagnosis, and day 1 is the day of inclusion. See page 18 for methods to maximize small sampling for children.

Table 2. Schedule of assessments for pediatric case patients.

| **Visit** | **Routine** | **Inclusion** | **Follow-up** | | | | | |
|---|---|---|---|---|---|---|---|---|
| **Visit number** | | **1** | **1.1*** | **2** | **2.1*** | **3** | **4** | **5** |
| **Timeline (day 0 = day of diagnostic test)** | | 1 | 3 | 7 | 10 | 14 | 28 | 56 |
| **Window period (days)** | | -1,+2 | ±1 | ±2 | ±1 | ±3 | ±7 | ±14 |
| Informed consent | | X | | | | | | |
| Entry criteria | | X | | | | | | |
| Physical exam | | X | | (X) | | (X) | | (X) |
| Clinical symptoms severity evaluation | | X | | X | | X | X | X |
| Chest imaging (X-ray ± CT) | X | (X) | | (X) | | (X) | | |
| Nasopharyngeal swab | X | X | X | X | (X) | X | X | (X) |
| Nasal wash** | | | (X) | (X) | (X) | (X) | (X) | (X) |
| Bronchoalveolar lavage specimen collection*** | (X) | (X) | (X) | (X) | (X) | (X) | (X) | |

| | | | | | | | | |
|---|---|---|---|---|---|---|---|---|
| Sputum specimen collection§ | | X | X | X | (X) | X | X | (X) |
| Other body fluid (tears, vesicles, stools) only if clinically appropriate | (X) | (X) | (X) | (X) | (X) | (X) | (X) | |
| Complete blood count | 2 | 2 | (2) | 2 | (2) | 2 | 2 | 2 |
| Blood chemistry (NaCl, BUN, creatinine, AST, ALT, alkaline phosphatase, CRP) | 2 | 2 | | (2) | | (2) | (2) | |
| Plasma for viral load and innate responses | | 2 | 2 | 2 | 2 | 2 | 2 | 2 |
| Blood for innate cell phenotyping | | 2 | 2 | 2 | 2 | 2 | 2 | 2 |
| Blood RNA preservation | | 1 | 1 | 1 | | 1 | 1 | 1 |
| Serum collection (antibody assessment)§§ | | 1-3 | 1-3 | 1-3 | | 1-3 | 1-3 | 1-3 |
| Cellular immune response (PBMCs)§§ | | 5-20 | | 5-20 | | 5-20 | 5-20 | 5-20 |
| Maximum blood volume (mL) | | 15-32 | 6-8 | 13-30 | 4 | 13-30 | 13-30 | 13-30 |
| Maximum cumulative blood volume (mL) | | 15-32 | 21-40 | 34-70 | 38-74 | 51-104 | 64-134 | **77-164** |

*Specimen collection will occur on D3 and D10 only for patients who are still hospitalized.
**Nasal wash may be offered with/as an alternative to nasopharyngeal swabbing in participants who may otherwise refuse the latter.
***Repeat specimen collection will occur only for patients who are still hospitalized and if prior samples have not already normalized (undetectable virus, blood count normalized, etc.).
(X) Only if performed/justified for clinical reasons
§ Only if productive cough, for viral load, innate cytokines and for detection of IgA & IgG antibodies.
§§ According to maximal age-based allowances, see below.

**To maximize small sampling for children, the following procedures will be used:**

- we will keep the plasma from the initial step of PBMCs separation for certain serological analyses, and the red cell pack for DNA extraction.
- we will take only 1ml of blood for blood RNA
- we will prioritize samples (based on age/weight) so as not to exceed the maximum allowance (see below).

The maximum blood volumes harvested for each visit will be in accordance with the European Regulation No 536/2014 on clinical trials on medicinal products for human use (18.9.2017), that states that “per individual, the trial-related blood loss should not exceed 3% of the total blood volume during a period of four weeks and should not exceed 1% at any single time. “

This means that research-related blood harvest will not exceed the following volumes:

| **Body weight (kg)** | **Circulating total blood volume (ml)** | **Maximum allowable sample volume over 4 weeks (ml) - 3% of total blood volume** | **Maximum allowable sample volume at single time (ml) - 1% of total blood volume** |
|---|---|---|---|
| 0.5 - 1.5 | 50 - 150 | 1.5 -4.5 | 0.5 – 1.5 |
| 2.5 - 5 | 250 - 500 | 7.5 – 15 | 2.5 – 5 |
| 5 - 12 | 480 - 960 | 14.4 – 28.8 | 4.8 - 9.6 |
| 12 - 20 | 960 - 1600 | 28.8 – 48 | 9.6 -16 |
| 20 - 30 | 1600 - 2400 | 48 – 72 | 16 – 24 |
| 30 - 70 | 2400 - 5600 | 48 – 168 | 24 – 56 |

Table: Maximum allowable research-related blood sample volumes. Total blood volume is approximately 80-90 ml/kg body weight, in neonates approximately 100 ml/kg body weight. Of note: when routine health care requires significant blood sampling, these maximums may even be excessive.

If blood sampling is taken for clinical purpose at additional time points, additional volume of blood will be taken simultaneously for the study, but not more than the amount allowed according to the child's weight per day (1% of blood volume, see table).

### 3.3.3 Study visits for adult household contacts

Household contacts will be followed closely in the two weeks following the index patient's diagnosis/suspected diagnosis and may be invited to return for antibody sampling at four weeks. If SARS-CoV-2 PCR becomes positive at any point, contacts will move, if providing informed consent, to the group of COVID-19 patients and be followed as indicated above. Day 0 is the day of the index patient's diagnosis/suspected diagnosis, and day 1 is the day of inclusion. Note that none of the assessments below are performed routinely as long as contacts remain asymptomatic; all will be conducted in the context of the study.

Table 3. Schedule of assessments for adult household contacts.

| **Visit** | **Inclusion** | **Follow-up** | | | | |
|---|---|---|---|---|---|---|
| **Visit number** | **1** | **2** | **3** | **4** | **5** | **6§** |
| **Timeline (day 0 = day of index patient's diagnosis/suspected diagnosis)** | 1 | 3 | 7 | 10 | 14 | 28 |
| **Window period (days)** | -1,+2 | ±1 | ±2 | ±2 | ±3 | +14 |
| Informed consent | X | | | | | |
| Entry criteria | X | | | | | |
| Physical exam | X | | (X) | | (X) | |
| Nasopharyngeal swab | X | X | X | X | X | |
| Nasal wash* | | (X) | (X) | (X) | (X) | |
| Complete blood count | 1 | 2 | 2 | 2 | 2 | |
| Blood chemistry (NaCl, BUN, creatinine, AST, ALT, alkaline phosphatase, CRP) | 2 | | | | | |
| HLA phenotyping for T cell responses | 1 | | | | | |
| Plasma for viral load and innate responses | 2 | 2 | 2 | 2 | 2 | |
| Blood for innate cell phenotyping | 2 | 2 | 2 | 2 | 2 | |
| Blood RNA preservation | 2 | 2 | 2 | ** | 2 | |

| | | | | | | |
|---|---|---|---|---|---|---|
| Serum collection (antibody assessment) | 6 | 6 | 6 | | 6 | 6 |
| Cellular immune response (PBMCs) | 32 | | 32 | | 32 | |
| Maximum blood volume (mL) | 48 | 14 | 46 | 6 | 46 | 6 |
| Maximum cumulative blood volume (mL) | 48 | 62 | 108 | 114 | 160 | 166 |

(X): only if symptomatic, and at any time if symptomatic
* Nasal wash may be offered with/as an alternative to nasopharyngeal swabbing for participants who may otherwise refuse the latter.
**If the patient's swab from day 7 becomes positive and on day 10 the participant provides consent to participate as a patient, one Paxgene tube (2 ml) will be drawn.
§ This visit will occur only for contacts whose PCR and serology tests were negative despite clinical or epidemiologic suspicion for COVID-19.

*Visit 1, Enrollment (Day 1)*

This visit will occur on day 1, with day 0 being the day of the index patient's diagnosis/suspected diagnosis. The following activities will occur during the first visit:

- The informed consent form (ICF) will be provided as described above; no further activity will take place until signing of the ICF
- Inclusion and exclusion criteria will be evaluated
- A targeted physical examination will be performed
- Nasopharyngeal swab for assessment of viral load (RNA)
- Nasal wash may be offered with/as an alternative to nasopharyngeal swabbing in participants who may otherwise refuse the latter

- <u>Blood specimen collection</u>:
    - Blood will be collected as indicated in Table 2 for:
        - Complete blood count and chemistry (electrolytes, renal and liver function)
        - Routine inflammatory markers
        - HLA phenotyping for further assessment of T cell responses
        - Plasma viral load quantification
        - Characterization of innate responses (plasma) and blood RNA preservation
        - Assessment of the presence and concentrations of antibodies to SARS-CoV-2 or other viral pathogens
        - Assessment of cellular adapted immune responses (isolated PBMCs), as appropriate:
            - Characterization of innate and adaptive immune responses by flow cytometry and ELISpot (i.e. magnitude, activation status, functionality, antigen-specificity) and assess their change over time in individuals in whom SARS-CoV-2 PCR may become positive
            - Identifying immune biomarkers during the early phase that can predict disease severity
        - Freezing and storage of serum and PBMCs for further characterization if the household contact becomes SARS-CoV-2 positive

*Visit 2 (Day 3 [±1])*

The following will occur/be collected:

- Nasopharyngeal swab
- Harvesting of blood for complete blood count, inflammatory markers, plasma viral load and innate responses, innate cell phenotyping, RNA preservation, and antibody assessment

*Visit 3 (Day 7 [±2])*

The following will occur/be collected:

- Nasopharyngeal swab
- Targeted physical exam if appropriate
- Harvesting of blood for complete blood count, inflammatory markers, plasma viral load and innate responses, innate cell phenotyping, RNA preservation, and antibody assessment

*Visit 4 (Day 10 [±2])*

The following will occur/be collected:

- Nasopharyngeal swab
- Harvesting of blood for complete blood count, inflammatory markers, plasma viral load and innate responses, innate cell phenotyping

*Visit 5 (Day 14 [±3])*

The following will occur/be collected:

- A targeted physical exam
- Nasopharyngeal swab
- Harvesting of blood for complete blood count, inflammatory markers, plasma viral load and innate responses, innate cell phenotyping, RNA preservation, and antibody assessment

*Visit 6 (Day 28 [+14])*

This visit will occur only for contacts whose PCR and serology tests were negative despite clinical or epidemiologic suspicion for COVID-19. Its purpose will be sampling for seropositivity at least four weeks after probable exposure to determine whether an asymptomatic (or pauci-symptomatic) infection occurred and thus to avoid misclassification.

<u>3.3.4 Study visits for pediatric household contacts</u>

Visits for pediatric household contacts will largely follow those of adult contacts except for decreased blood volume collection: PBMCs will be collected only if appropriate for body weight (see Table 4). See page 18 for methods to maximize small sampling for children. If SARS-CoV-2 PCR becomes positive at any point, contacts will move, provided informed consent, to the group of COVID-19 patients and be followed as indicated above. Day 0 is the day of the index patient's diagnosis/suspected diagnosis. Note that none of the assessments below are performed routinely as long as contacts remain asymptomatic; all will be conducted in the context of the study.

Table 4. Schedule of assessments for pediatric household contacts.

| Visit | Inclusion | Follow-up | | | |
|---|---|---|---|---|---|
| **Visit number** | **1** | **2** | **3** | **4** | **5*** |
| **Timeline (day 0 = day of index patient's diagnosis/suspected diagnosis)** | 1 | 3 | 7 | 14 | 28 |
| **Window period (days)** | -1,+2 | ±1 | ±2 | ±3 | +14 |
| Informed consent | X | | | | |
| Entry criteria | X | | | | |
| Physical exam | X | | (X) | (X) | |
| Nasopharyngeal swab | X | X | X | X | |
| Nasal wash** | (X) | (X) | (X) | (X) | |
| Complete blood count | 2 | 2 | 2 | 2 | |
| Plasma for viral load and innate responses | 2 | 2 | 2 | 2 | |
| Blood for innate cell phenotyping | 2 | 2 | 2 | 2 | |
| Blood RNA preservation | 1 | 1 | 1 | 1 | |
| Serum collection (antibody assessment) | 1-3 | 1-3 | 1-3 | 1-3 | 1-3 |
| Cellular immune response (PBMCs)* | 5-20 | | 5-20 | 5-20 | |
| Maximum blood volume (mL) | 13-30 | 8-11 | 13-30 | 13-30 | 1-3 |
| Maximum cumulative blood volume (mL) | 13-30 | 21-41 | 34-71 | 47-101 | 48-104 |

(X): only if symptomatic, and at any time if symptomatic
* This visit will occur only for contacts whose PCR and serology tests were negative despite clinical or epidemiologic suspicion for COVID-19.
*** Nasal wash may be offered with/as an alternative to nasopharyngeal swabbing in participants who may otherwise refuse the latter.

The same rules will be followed for maximal blood harvesting as indicated for infected patients (see above).

### 3.4 Withdrawal and discontinuation

In accordance with the principles of the current revision of the Declaration of Helsinki, a participant has the right to withdraw from the study at any time and for any reason, and is not obliged to give his or her reason(s) for doing so. The Investigator may withdraw the participant at any time in the interests of the participant's health and well-being. In addition, the participant may withdraw/be withdrawn for any of the following reasons:

- Ineligibility (either arising during the study or retrospectively, having been overlooked at the first visit)
- Significant protocol deviation: *e.g.*, failure to obtain informed consent prior to any study-specific test or procedure
- For participants whose diagnosis could not be laboratory-confirmed and whose symptoms ultimately were attributed by the attending physician to an alternate diagnosis

The reason for withdrawal will be recorded in the CRF. If a participant withdraws from the study, data and samples collected before his/her withdrawal from the study will be used/stored unless the participant specifically requests otherwise.

Every effort will be made by the Investigator to keep the participant in the study. If a participant has to be withdrawn, all efforts will be made to complete and report the study observations as thoroughly as possible.

When a participant withdraws from the study before the planned end of the study period, all investigations scheduled for the end-of-study visit should be performed if the participant agrees. The end-of-study evaluation will be completed at the time of the participant's withdrawal, with an explanation of the reason for this entered into the respective "end-of-study" section of the CRF as follows:

- Protocol violation (specify)
- Medical condition (e.g., bleeding diathesis)
- Consent withdrawal
- Adverse event (in this case, phlebotomy-related)
- Death
- Other (specify)

# 4 STATISTICS, METHODOLOGY AND LABORATORY ANALYSES

## 4.1. Statistical analysis plan

### 4.1.1 Sample size

The sample size will not be calculated; the size of this convenience sample will be determined by the number of patients diagnosed and willing to participate, and by the level of funding study investigators can secure. At the time of submission, at least 50 patients plus their household contacts (maximum number 250) are expected to be included.

### 4.1.2 Statistical analysis plan

Descriptive statistics will be employed, with comparisons between groups using the independent Student's t-test for continuous data and a chi-square or Fisher's exact test for categorical data, as appropriate. Univariate and multivariate logistic and linear regression models will be constructed to evaluate associations between viral load quantities (plasma and respiratory samples), the kinetics of viral load (*i.e.*, slope of ascent or descent), symptom burden, and overall clinical outcome. Logistic and linear regression will also be used to evaluate associations between demographic, clinical and biological/immunological information and antibody titers (seropositivity, other specific titer thresholds [categorical], antibody titers [continuous]). For secondary immunogenicity endpoints, descriptive summaries and plots over the time course for both individual patients and aggregated results will be presented. Where appropriate, highly skewed data will be log-transformed and presented as geometric means with 95% confidence intervals.

Should there be no or minimal immune response in some infected subjects, logistic regression will be used to evaluate associations between non-persistence and baseline demographic, biological and immunologic features, such as HLA type, early T-cell and B-cell responses and functionality, inflammatory cytokine production, etc.

Multiparametric tests including dimension reduction, distances matrices and supervised clustering, can be applied to elucidate the putative links between the different readouts with the severity of the disease and/or the pre-existing immunity to circulating CoVs.

Associations with P values of ≤.05 (two-sided) will be considered statistically significant.

#### 4.1.3 Specific immunological analyses

The analyses will be described in terms of the proportions of responders and the magnitude of response over time:

- SARS-CoV2-specific IgG, IgA and IgM antibody titers in serum and sputum measured by ELISA or rIFA will be calculated at all timepoints. Geometric mean titers (GMT) and 95% confidence intervals will be calculated for each time point. Antibody titers will continue to be displayed as reverse cumulative distribution (RCD) curves to illustrate antibody distribution. Comparisons between time points will be performed by the Kruskal-Wallis test. Seropositivity rates will be assessed at each time point and reported with 95% confidence intervals. Mean fold increase or decrease will be assessed.
- Neutralizing antibody titers will be measured in serum and sputum samples using PRNT and/or MNT at all time points.
- The change in the kinetics, phenotype and functionality of B cell, CD4 and CD8 T cell responses (adaptive immune responses) by ELISpot and flow cytometry will be assessed during the phase of infection on days 0, 7, 14 and at day 28 after symptom onset. For these descriptive endpoints, we will generate summary statistics, and visualize the data using histograms and boxplots when applicable. We will test for changes over baseline and recovery using a two-sided paired t-test or Wilcoxon signed-rank test. We will determine which test to use by assessing whether the differences between pairs are normally distributed. The tentative threshold for significance is p-value ≤ 0.05.
- The immunoglobulin repertoire, avidity and protective capacity of monoclonal antibodies in a subset of 5-10 subjects. We will generate summary statistics, and visualize the data using histograms and boxplots.
- Data from RNA sequencing of immune cell subsets will be analyzed using the statistical software R and other appropriate methods, either by stand-alone software or modules in the R framework. Unsupervised learning techniques such as principal component analysis (PCA) and clustering will be used for the visualization and identification of global patterns.
- mRNA levels in whole blood will be measured by RNAseq. Reads will be mapped on the latest version of the human genome (GRCh38) and only reads mapping uniquely to the genome will be considered for the rest of the analysis. After normalization, changes in gene expression between groups will be assessed by Generalized Linear Model (GLM). Up- or down-regulated immune pathways will be performed by appropriate analysis, such as Gene Set Enrichment Analysis (GSEA).

#### 4.1.4 Specific virologic analyses

The analysis will be described in terms of quantified viral load per specimen (viral copies/ml of original material), presence/absence of infectious virus over time and whole genome sequence information of virus isolates and/or patient samples:

- Testing for SARS-CoV-2 will be performed by quantitative real-time RT-PCR (E-gene screening and RdRP-gene confirmation assay for initial confirmation of the case, for all later specimens only E-gene assay and quantification of viral load by synthetic control)
- Testing for presence of infectious virus will be performed by inoculating VeroE6 cell lines with patient material and daily microcopy for assessment for cytopathic effect. In case of visible CPE of cells, confirmation of SARS-CoV-2 RNA in supernatant of initial inoculation on day 0 and on day of CPE observation.
- Whole genome information of clinical specimens and virus isolates will be done by whole genome sequencing either by Next-generation sequencing or by conventional PCR followed by amplicon sequencing of obtained PCR products. Phylogenetic analysis will be performed through an already existing bioinformatics platform.

### 4.2. Handling of missing data

Missing data for the primary outcome will be reported as such; sensitivity analyses with worst- and best-case scenarios will be used to explore the range of possible outcomes were those data available. If missing data for the primary outcome exceed 5%, multiple imputation may be employed. At regular intervals, the total number of study participants and the level of adequate follow-up will be assessed; depending on the level of missing data and the evolution of the outbreak, prolongation of recruitment for compensation/replacement may be explored as needed.

### 4.3. Potential biases and risk mitigation strategies

Factors that may bias the results of observational studies include:[8]

1. **Selection biases** resulting from the way study subjects are recruited arise when the study population is not a random selection from the target population. In our study, this mainly refers to the severity of symptoms, as the participation of hospitalized patients with more severe disease could be easier to obtain than those of mildly sick home-cared patients. The participation of adult household contacts is likely to be higher than those of small children. To mitigate this risk, we will keep a list of each potential participant so as to know the rate of refusal among adult vs pediatric cases vs controls. Furthermore, our study design including pauci-symptomatic healthcare workers and non/pauci/mildly symptomatic secondary cases should ensure sufficient variability in disease severity. Lastly, the available information is currently so limited that this cohort study could really make a difference.
2. **Differing rates of study participation** depending on the subjects' cultural background, age, or socioeconomic status will also occur. The risk mitigation approaches will be the same as above.
3. **Information bias** results from wrong or inexact recording of individual factors, either risk factors or the disease being studied. As we will study an acute disease at a specific point in time, this should not generate significant biases in our study.
4. Measurement errors may occur as the time between sampling and processing will be shorter for hospitalized than home-based patients. We will mitigate this risk by transporting specimen on cold-packs from the homes to the laboratories.
5. Confounders are to be expected in an observational study: we may not capture important determinants of disease severity. This will be assessed by splitting the group into subgroups (strata) defined by different levels of potential confounders (age, habits, etc.). Analyses will first be performed unstratified and then within the individual strata, and the Mantel-Haenszel

estimate used to combine the individual effect estimates from the stratified evaluation, correcting for the confounder.

# 5 OBSERVATIONAL SUBSTUDIES

## 5.1 Nested prospective observational cohort study on cytokine responses and viral load in nasal wash

### 5.1.1 Background and rationale

Local immune responses, in particular cytokine levels, have been previously recognized to be associated with clinical course during respiratory viral infection.[9-11] Although age and co-morbidities have been identified as negative prognostic factors during COVID-19 infection,[12] there is a wide range of COVID-19 clinical manifestation among young patients without co-morbidities. While the majority will have a flu-like syndrome, a small proportion will develop pneumonia and eventually need hospital admission for oxygen support. To date, there are no data on the local immune response in the upper respiratory tract and the potential correlation with COVID-19 level of disease severity. Nasal wash is a simple and non-traumatic procedure commonly used to obtain samples from the nasopharynx.[13] The procedure is deemed to be more sensitive for collecting the mucosal lining fluid, rich in cytokines, than the nasopharyngeal swabs routinely performed for diagnostic tests.[10, 13] As described above, we aim to understand the local immune response mounted in the upper respiratory tract during the early phase of SARS-Cov-2 infection and to assess correlation with local viral load; specimens collected via nasal wash may yield more sensitive results.

### 5.1.2 Nested nasal-wash study design, setting and population

All patients with SARS-CoV-2 infection included in the master study "Understanding COVID-19" who can perform a nasal wash and, if possible, have had symptom onset within 5 days of their diagnosis, will be eligible for this nested study. Contacts of patients will also be eligible for nasal-wash sampling; they will be analyzed separately as long as SARS-CoV-2-negative. The nasal wash will be performed only until fever resolution during planned visits of the main study.

Enrollment will end after 15 patients have been included. The size of this convenience sample has not been calculated, but is based upon prior experience described in the literature.[10, 13] No household contact will be included in the nested study unless they test positive and switch to the case cohort.

Nasal wash will be performed according to the institutional protocol (see https://www.youtube.com/watch?v=3cMoR7hSPF8&feature=youtu.be) and adapted from previous publications.[14, 15] Briefly, 3 ml of NaCl 0.9% are injected in the nose for nasal wash and regurgitated. 2 ml are then retrieved and mixed with 1 ml of DMEM for nucleic acid preservation.

Nasal wash will be used to measure cytokines, viral loads, antibodies and immune cells:

Cytokines will be measured by a multiplex technology (Luminex) containing 24 analytes (similar to the method used in the main study). The virus will be inactivated or samples proven to be RT-PCR negative before proceeding to the measurement in supernatants.

If relevant, and depending on the timing of sample collection, exploratory analysis of SARS-CoV-2 -specific IgG and IgA antibody titers and immune cells will be performed in nasal washes using the same methods used in the main study.

Viral load will be determined by one of the RT-PCR assays routinely used at the Laboratory of Virology.

5.1.3 Nested nasal-wash study outcome

The primary outcome is the assessment of the cytokine response in the nasopharynx during the early phase of COVID-19 infection.

The secondary outcomes are:
- To determine the kinetics of local cytokine response and viral titers in the nasopharynx until resolution of symptoms
- To assess the level of SARS-CoV-2-specific antibodies in nasal wash
- To assess the relationship between SARS-CoV-2 viral titer, cytokine levels and SARS-CoV-2 specific antibodies in nasal wash
- To compare the levels of cytokine between nasal wash and in blood samples (see master study)

5.1.4 Nested nasal-wash study statistical considerations

Viral titer and cytokine levels will be expressed individually and in median values with interquartile range. The Mann-Whitney test will be used to assess any differences in median cytokine titers between nasal wash and blood samples. The Kruskal-Wallis test will be used to assess any differences in the cytokine and viral titers among tested time points. The significance level will be two-sided $\alpha > 0.05$. Spearman's non-parametric correlation test will be used to assess the correlation between viral titer and cytokine levels in respiratory samples. If applicable, we will add exploratory data integration with clinical symptoms and evolution.

5.1.5 Nested study structure

Dr. Diem-Lan Vu Cantero will be the coordinator of this nested study.

**5. 2 Substudy: PERSISTENCE of humoral and cellular immunity against SARS-CoV-2**

5.2.1 Background and rationale for the PERSISTENCE substudy

Emerging data currently show that patients infected with SARS-CoV-2 develop IgM and IgG antibodies[16, 17] but little is known about the development of cell-mediated immunity. It is also unknown whether humoral and/or cellular immunity are good correlates of protection against reinfection. Immunity waning after exposure to other coronaviruses such as SARS-CoV-1 has been observed[18] and is expected to occur sooner or later – and to directly impact infection-induced protection, or lack thereof. Therefore, there is a need to evaluate the persistence of humoral and cellular immunity among patients infected with SARS-CoV-2 as well as their correlation, if any, with protection against clinical reinfection.

5.2.2 The PERSISTENCE substudy design, setting and population

To facilitate the logistics of this prospective observational longitudinal substudy, patients diagnosed with SARS-CoV-2 infection will be identified by occupational medicine through the hospital's surveillance network, which includes laboratory notification of specimens positive for SARS-CoV-2 testing. If not hospitalized or present at time of diagnosis, they will initially be contacted by telephone or email.

Inclusion criteria are (1) currently employed at HUG; (2) age 18 years old or above; (3) laboratory-confirmed SARS-CoV-2 infection. The only exclusion criterion is the inability to provide informed consent.

This substudy's particular focus on long-term immunity means that it may also include participants who are not necessarily already taking part in the ongoing observational "master study" described above, which includes a heavy schedule of assessments over only a short period (five visits over two months). In an effort to avoid asking for too many visits from any one individual, the Persistence substudy will mostly not include persons who have already agreed to participate in the "master study". Yet in the spirit of efficiency and cooperation, the substudy will leverage the infrastructure that has already been built by the master study: the clinical and laboratory teams and pathways, the electronic CRF, etc. Most importantly, it will include the exact same immunological analyses, *i.e.,* is really a part of "UNDERSTANDING COVID".

The substudy's specific population of hospital workers has been chosen for the sake of pragmatism: these individuals are likely to be easily reachable for the relatively longer duration of the project, and follow-up visits will be, we hope, less cumbersome for them, since they will occur at their place of work and thus require little extra effort and less planning.

Enrolled participants will also have five study visits, but at different time points from those of the master study.These visits will consist of one blood draw at the following time points after SARS-CoV-2 diagnosis (day 0): day 28 (-1, +2 weeks), month 3 (±2 weeks), month 6 (±3 weeks), month 12 (± 4 weeks) and month 24 (± 6 weeks). Please see the table below for more details. The visits will take place at the HUG.

The PERSISTENCE substudy will start in April 2020 and will continue for two years following the enrolment of the last participant.

| **Visit** | **Inclusion** | **Follow-up** | | | | |
|---|---|---|---|---|---|---|
| **Visit number** | * | **1** | **2** | **3** | **4** | **5** |
| **Timeline (day 0 = day of diagnosis)** | 0 | Day 28 | Month 3 | M6 | M12 | M24 |
| **Window period** | +28 days | -7, +14 d | ±2 weeks | ±3 weeks | ±4 weeks | ±6 weeks |
| Informed consent | X | | | | | |
| Entry criteria | X | | | | | |
| Serum collection (antibody assessment) | | 6 ml | 6 ml | 6 ml | 6 ml | 6 ml |
| Cellular immune responses (PBMCs) | | 32 ml | - | 32 ml | 32 ml | 32 ml |
| HLA phenotyping for T cell responses | | 2 ml | - | - | - | - |
| Maximal blood volume | | 40 ml | 6 ml | 38 ml | 38 ml | 38 ml |
| Maximal cumulative blood volume | | 40 ml | 46 ml | 84 ml | 122 ml | 160 ml |

*Nota bene*: As a high number of COVID+ hospital workers were diagnosed between March 15th and March 30th, we may contact these individuals up to 6 weeks after diagnosis, as long as visit 1 can still happen in the indicated time-frame. This will allow us to increase the number of eligible study participants without influencing the design, as the first visit occurs only four weeks (±2) after diagnosis.

5.2.3 Study outcomes of the PERSISTENCE substudy

In accordance with the primary aim of the UNDERSTANDING COVID-19 study, the primary aim of this prospective observational study is to evaluate the persistence of humoral immunity at 2 years after SARS-CoV-2 infection among hospital workers, as defined by the GMT of total IgG antibodies to SARS-CoV-2 assessed by ELISA.

In accordance with the UNDERSTANDING COVID-19 study, secondary outcomes are:
-The GMT of total IgG antibodies to SARS-CoV-2 assessed by ELISA at 1,3,6, and 12 months after diagnosis.

-The GMT of total IgA and IgM antibodies to SARS-CoV-2 assessed by ELISA at 1,3,6, 12 and 24 months after diagnosis.
-The persistence of T- and B-cell immunity, defined as the percentage of SARS-CoV-2-specific T- and B-cells, respectively, up to 2 years after SARS-CoV-2 infection at 1, 6, 12 and 24 months, in a random subset of participants if allowed by funding.
-The identification of potential risk factors for waning of humoral and cellular immunity (*e.g.*, age, gender, comorbidities).
- The proportion of hospital workers who experience symptomatic SARS-CoV-2 reinfection at 12 and 24 months after the first infection.

Additional exploratory aims are:
-The impact of symptomatic SARS-CoV-2 reinfection on IgG antibody titers and B-cell and T-cell responses.
-The proportion of unidentified re-infection, as evidenced by an increase in IgG antibodies, B- and T-cell responses.
-Potentially, to identify correlates of protection against reinfection, which could be used as potential markers of vaccine efficacy.

### 5.2.4 Laboratoy methods for the PERSISTENCE substudy

For this substudy, the laboratory methods will be the same as those used for the ongoing observational “master study”.

### 5.2.5 Statistical considerations for the PERSISTENCE substudy

For this substudy there will be no formal sample size calculation. Instead, we will enroll a convenience sample of as many hospital workers as possible until the end of the epidemic. Given the number of previously infected employees, we plan to enroll 200 participants.
Descriptive statistics will be employed. Logistic and linear regression models will be constructed to evaluate associations between immunoglobulin levels (or their presence/absence), age, gender and comorbidities. Where appropriate, highly skewed data will be log-transformed and presented as geometric means with 95% confidence intervals. Associations with P values of ≤.05 (two-sided) will be considered statistically significant.

### 5.2.6 Ethical considerations for the PERSISTENCE substudy

The PERSISTENCE substudy ICF will be made available to hospital workers in advance such that they have ample time and opportunity to inquire about details of the study and to decide whether or not to participate in the study. Consenting participants must sign and date the ICF before any study-specific procedures may be performed.

### 5.2.7 Data handling for PERSISTENCE substudy

For each enrolled hospital worker, a CRF in Teleform will be used. Participants will be identified by means of a study number and no personal information will be transcribed in the CRF. All documents will be password-protected. Collected biological samples will also be coded.

### 5.2.8 Substudy structure

Dr. Arnaud L’Huillier will be the coordinator of the PERSISTENCE substudy.

# 6 REGULATORY ASPECTS AND SAFETY

## 6.1 Local regulations / Declaration of Helsinki

This research project will be conducted in accordance with the protocol, the Declaration of Helsinki [3], the principles of Good Clinical Practice, the Human Research Act (HRA) and the Human Research Ordinance (HRO) [1] as well as other locally relevant regulations. The Project Leader acknowledges her responsibilities as both the Project Leader and the Sponsor.

## 6.2 Notification of safety and protective measures (HRO Art. 20)

The Project Leader is promptly notified (within 24 hours) if immediate safety and protective measures have to be taken during the conduct of the research project. The Ethics Committee will be notified via BASEC of these measures and of the circumstances necessitating them within 7 days.

## 6.3 Serious events (HRO Art. 21)

If a serious event occurs, the research project will be interrupted and the Ethics Committee notified on the circumstances via BASEC within 7 days according to HRO Art. 21[1].

## 6.4 Biosafety procedures

### 6.4.1 Personnel safety during study visits

The Standard Operating Procedure for Minimizing Exposure During Study Visits (Annex 1) describes in detail the steps that will be taken to minimize exposure of study personnel and contacts to SARS-CoV-2 during study visits. The main principles are these:

- All collaborators will be required to undergo formal training before conducting a house call or any inpatient visit
- On his/her first home visit and first inpatient visit, the collaborator will be accompanied by a collaborator trained on infection-control measures when coming into contact with SARS-CoV-2+ patients
- The collaborator will first conduct assessments on all contacts (starting with asymptomatic ones) while the patient remains in his room
- The contacts will then move to their rooms while the nurse prepares to enter the patient's room

See the SOP in annex for more details.

### 6.4.2 Laboratory biosafety

Research assays involving live virus will be performed exclusively in the Biosafety Level (BSL)-3 laboratory, as is routine practice. Studies have found no[19] or only very low copy numbers of viral RNA (cycle threshold value >35)[20] in serum samples of infected patients. It has been shown in respiratory samples that there was no isolation of infectious particles if RNA copy numbers were below $10^6$ copies/ml. These findings

[1] A serious event is defined as any adverse event where it cannot be excluded, that the event is attributable to the sampling of biological material or the collection of health-related personal data, and which:
a. requires inpatient treatment not envisaged in the protocol or extends a current hospital stay;
b. results in permanent or significant incapacity or disability; or
c. is life-threatening or results in death.

indicate that even if RNA can be found in blood samples no infectious virus is expected and these samples do not pose a risk for laboratory staff. Nevertheless, precautions will be taken as follows:

**Blood specimen (includes PBMC, serum and plasma):**
We will quantify viral RNA in a subset of serum samples from mild, severe and critical cases of COVID-19 to determine whether these results can be confirmed in our Geneva patient cohort. To be on the safe side, all samples with viral RNA copy numbers above $10^5$ copies/ml, will be considered as potentially infectious and we will process like diagnostic specimens in the P2 laboratory at the HUG until proper inactivation by a proven method (*e.g.*, formaldehyde, beta-propiolactone treatment or gamma irradiation).

**Respiratory and fecal samples:**
For the detection of viral RNA load by qRT-PCR in fecal or respiratory samples, we will use standard procedures established for the diagnosis of patients in the virology laboratory at the HUG. For the detection of antibodies or cytokines in respiratory samples, we will treat samples either with beta-propiolactone or gamma-irradiate samples using a gamma source at the CMU. To confirm that our procedures completely inactivate SARS-CoV-2, we will determine viral titers in respiratory samples spiked with known concentrations of SARS-CoV-2 (comparable to concentrations found in patient samples) using plaque assay.

### 6.5 Amendments

Substantial changes to the project set-up, the protocol and relevant project documents will be submitted to the Ethics Committee for approval according to HRO Art. 18 before implementation. Exceptions are measures that have to be taken immediately in order to protect the participants.

### 6.6 End of project

Upon project completion or discontinuation, the Ethics Committee is notified within 90 days. As described in the informed consent forms, coded biological samples will be stored in HUG and/or UNIGE Faculty of Medicine laboratories for up to 15 years after end of study. The data generated throughout the course of the study will remain coded and will be owned by the study Sponsor (see below).

### 6.7 Insurance

In the event of project-related damage or injuries, the liability of the HUG provides compensation per current protocol, except for claims that arise from misconduct or gross negligence.

## 7 FURTHER ASPECTS

### 7.1 Overall ethical considerations

This study will provide valuable information for future patients, their physicians and societies, which have come to a standstill because of SARS-CoV-2. There currently is little understanding as to why children experience almost no clinical illness and young, healthy adults relatively little after exposure to this virus. The study will characterize immune responses and viral behavior in these populations, and contrast them with those in older patients and others with higher symptom severity. The identification of protective factors will be key for selecting targets for clinical-therapeutic applications in the coming months.

Given the current costs of this virus to entire populations on six continents—costs that are being exacted from individuals in myriad ways, whether they be health/mortality-related, economic, psychic—we believe that the overall social and scientific value of this study will be considerable, and that there is an overall fair balance for patients and contacts who wish to participate.

Investigators pledge to keep participants and the medico-scientific community aware of any novel and/or incidental findings as quickly as they can be verified and vouched for by the Project Leader.

No human genetic analyses are planned: only human leukocyte antigen (HLA) screening will be performed by DNA and solely viral RNA and protein analyses (RNA, amino-acid sequencing and characterization) will be performed.

### 7.2 Risk-Benefit Assessment

The major risk inherent to the study is the potential exposure or increased exposure of study/laboratory personnel and household contacts, respectively, to SARS-CoV-2+ patients; these are low within families and further mitigated as described in Annex 1 and Sections 5.4.1 and 5.4.2 above. The risks to participants are minimal and include the risks of bruising after blood draw and discomfort after swabbing. See immediately above for the overall risk-benefit assessment for patients, contacts, and society as a whole.

### 7.3 Rationale for the inclusion of vulnerable participants

The following categories of vulnerable patients will be included: children (0-13 years), adolescents (14-18 years), pregnant women, and persons unable to consent due to transient or long-term incapacity.

Clinically, young children are being unusually “spared” by this virus despite documented exposure (*e.g.*, both parents with clinical disease and observed shedding). Their immunologic responses and cellular interactions (receptor attachment, etc.) with the virus could provide key insights into viral mechanisms of cell entry, human cellular responses (apoptosis, etc.). This could both point to targets for antiviral therapies and prevention and inform about the adequacy of containment, school closure, etc.

There are almost no data available on pregnant women infected by this virus; pregnancy is a time of modified cellular immunity. The medical community needs to understand this population’s risk, if any, for increased transmission (to contacts and to the fetus) and for increased symptom severity/kinetics.

Elderly or sick patients are most at risk for COVID-related morbidity and mortality and are also the most likely to have reduced capacity. Excluding this population from the study would create an important selection bias that would obfuscate the larger clinico-immunological picture, all of which is necessary to deduce, ultimately, the factors that contribute to and the factors that protect from acquisition and progression of viral disease.

Patients with severe COVID-19 are hypoxic and thus may have a transient lack of capacity to provide informed consent. Excluding these patients, in the event that neither a next of kin nor a legal guardian is available, would create an important selection bias that could well jeopardize the validity of study results. These patients may thus be included provisionally provided their consent or the consent of their next of kin is sought without delay. *In any case, their data and samples will not be used unless they specifically provide informed consent later on*, once their medical condition has improved.

Informed consent for children and for patients without capacity will be obtained from their parents/next of kin/legal representatives (see ICF documents). If a patient without capacity long-term has no next of

kin or legal representative who can provide informed consent on his/her behalf, that patient will not be included in the study.

# 8 QUALITY CONTROL AND DATA PROTECTION

## 8.1 Quality measures

For quality assurance the Ethics Committee may visit the research sites. Direct access to the source data and all project related files and documents will be granted on such occasions.

*Internal monitoring*
Internal monitoring will be performed at regular intervals according to ICH Good Clinical Practice (GCP); results will be documented in the study's "Trial Master File" (TMF). In particular, source data verification will be performed to ensure consistent data collection practices and quality.

*Modification to protocol*
Investigators will not implement any deviation from, or changes to, the study protocol without mutual agreement AND in written form of an amendment to study protocol. The only exceptions are where it is necessary to eliminate an immediate hazard to study patients, or when the changes involve only logistical or administrative aspects of the study (*e.g.*, change of telephone number). Protocol amendments will be submitted to the CCER as described above. The Principal Investigator is responsible for ensuring that changes to an approved study, during the period for which CCER approval has already been given, are not initiated without CCER review and approval.

## 8.2 Data recording and source data

The Investigator will maintain appropriate medical and research records for this study, in compliance with ICH E6 GCP and regulatory and institutional requirements for the protection of confidentiality of participants. The Principal Investigator, co-investigators and clinical research nurses will have access to records. Investigators will permit authorized representatives of the Sponsor and the ethics committee to examine (and when required by applicable law, to copy) clinical records for the purposes of monitoring, quality assurance reviews, audits and evaluation of the study safety and progress.

All protocol-required information will be collected in source documents and entered into the electronic CRF (RedCAP®) by study investigators. Source documents are original documents, data, and records from which the patient's CRF data are obtained. For this study, these will include, but are not limited to, patient consent form, clinical notes (medical history, vital signs, physical examination, concomitant medications, etc.), imaging results, laboratory records, and correspondence. All source data will be stored securely. The information to be recorded in the CRF is listed in detail in Box 1.

Box 1. Participant information to be recorded in the CRF.

<u>Demographic data</u>
- Age, sex, ethnicity (self-reported)
- Household pets, other recent contact with animals
- Employment (frequent contact with children, adolescents, patients, etc.)

<u>Baseline clinical data</u>
- Detailed past and current medical history, if any

- Current medications, if any (with specific attention to anti-inflammatory / antipyretic medications)
- Relevant exposure history if known
- Habits (smoking, BMI, etc.)
- Recent (≤4 weeks) colds, flulike illnesses, other respiratory events

History of the present illness

- Clinical presentation (doctors', nurses', paramedics' written observations)
- Evolution of symptoms
- Physical findings (see schedule of assessments)
- Imaging results:
  - written results of X-rays, CT scans, MRI, or other imaging modalities
- Laboratory results:
  - full blood counts, chemistry, inflammation
  - immunoglobulin levels (if measured)
  - virology results (results of other viral screening tests)
  - other microbiology (bacteriologic, fungal) results, if any
  - any item that may be relevant to disease and progression

### 8.3 Confidentiality and coding

**Project data** will be handled with uttermost discretion and is only accessible to authorized personnel who require the data to fulfil their duties within the scope of the research project. On the CRFs and other project specific documents, participants are identified only by a unique participant study number. Included patients' and contacts' clinical data will be coded: upon identification of eligibility, each patient/contact will receive a study number so that no personally identifying information (PII) will be entered into the CRF or the study database. Thus on these and other project-specific documents, participants will be identified only by that unique participant number. Other project-specific documents will consist of those recording laboratory results (virology and immunology laboratories): these laboratories will receive the samples labeled only with the participant's study number, not with any PII, and will generate results according to the participant's study number.
The "crosswalk" table linking the patient's identifying information to his/her study number will be password-protected and kept by the Project Leader. The Redcap platform ensures traceability and safety. Data will be stored on HUG servers allowing for safety back-ups as needed.

**Biological material** in this project is not identified by participant name but by a unique participant number (see above). Biological material is appropriately stored in a restricted, locked area only accessible to authorized personnel. The same crosswalk table linking the patient's identifying information to the study number will apply to biological samples and will be password-protected and kept by the Project Leader. The storage freezers of the virology and immunology laboratories are temperature-monitored and temperature-secured. Inventory lists of all samples entering the storage facility and freezers will be kept by laboratory personnel and the Project Leader; these lists will be cross-checked with samples at scheduled intervals during the year. At this juncture, there is no plan to send biological samples abroad; should this change, a protocol amendment with modifications to the patients' and contacts' ICF will be submitted.

### 8.4 Retention and destruction of study data and biological material

Study data and biological material will be stored for 15 years after study termination. Thereafter, biological samples will be destroyed according to usual biosafety/laboratory protocols in place in the

virology and immunology laboratories, which include documentation of each sample and its destruction. There is currently no plan for further use of samples (biobanking).

## 9 FUNDING / PUBLICATION / DECLARATION OF INTEREST

The study is initially financed by an emergency grant from the Fondation privée des HUG, and the research funds of the Center for Vaccinology (University of Geneva) and the Centre for Emerging Viral Diseases (University of Geneva, HUG). Funding will be sought from other sources including through the SNF call for submissions on coronaviruses. Results will be submitted in the form of original articles to peer-reviewed, open-source international journals for the benefit of the medical and scientific communities. The Project Leader and all co-investigators declare no conflict of interest in the concept and execution of this study. Fully anonymized data that are not already published may be shared on reasonable request from a qualified investigator, at the discretion of the Project Leader.

## 10 REFERENCES

1. Wu Z, McGoogan JM. Characteristics of and Important Lessons From the Coronavirus Disease 2019 (COVID-19) Outbreak in China: Summary of a Report of 72314 Cases From the Chinese Center for Disease Control and Prevention. JAMA 2020.
2. European Centers for Disease Control and Prevention [online]. Available at: https://www.ecdc.europa.eu/en/novel-coronavirus-china.
3. National Early Warning Score (NEWS) 2 [online]. Available at: https://www.rcplondon.ac.uk/projects/outputs/national-early-warning-score-news-2.
4. Angus DC, Seymour CW, Coopersmith CM, et al. A Framework for the Development and Interpretation of Different Sepsis Definitions and Clinical Criteria. Crit Care Med 2016;44:e113-121.
5. Charlson M, Szatrowski TP, Peterson J, Gold J. Validation of a combined comorbidity index. J Clin Epidemiol 1994;47:1245-1251.
6. Lim WS, van der Eerden MM, Laing R, et al. Defining community acquired pneumonia severity on presentation to hospital: an international derivation and validation study. Thorax 2003;58:377-382.
7. Fine MJ, Auble TE, Yealy DM, et al. A prediction rule to identify low-risk patients with community-acquired pneumonia. N Engl J Med 1997;336:243-250.
8. Hammer GP, du Prel JB, Blettner M. Avoiding bias in observational studies: part 8 in a series of articles on evaluation of scientific publications. Dtsch Arztebl Int 2009;106:664-668.
9. Nicholson EG, Schlegel C, Garofalo RP, et al. Robust Cytokine and Chemokine Response in Nasopharyngeal Secretions: Association With Decreased Severity in Children With Physician Diagnosed Bronchiolitis. The Journal of infectious diseases 2016;214:649-655.
10. Hansel TT, Tunstall T, Trujillo-Torralbo MB, et al. A Comprehensive Evaluation of Nasal and Bronchial Cytokines and Chemokines Following Experimental Rhinovirus Infection in Allergic Asthma: Increased Interferons (IFN-gamma and IFN-lambda) and Type 2 Inflammation (IL-5 and IL-13). EBioMedicine 2017;19:128-138.
11. Diaz PV, Valdivia G, Gaggero AA, et al. Pro-Inflammatory Cytokines in Nasopharyngeal Aspirate From Hospitalized Children With Respiratory Syncytial Virus Infection With or Without Rhinovirus Bronchiolitis, and Use of the Cytokines as Predictors of Illness Severity. Medicine 2015;94:e1512.
12. Wang D, Hu B, Hu C, et al. Clinical Characteristics of 138 Hospitalized Patients With 2019 Novel Coronavirus-Infected Pneumonia in Wuhan, China. Jama 2020.

13. Jochems SP, Piddock K, Rylance J, et al. Novel Analysis of Immune Cells from Nasal Microbiopsy Demonstrates Reliable, Reproducible Data for Immune Populations, and Superior Cytokine Detection Compared to Nasal Wash. PloS one 2017;12:e0169805.
14. Gritzfeld JF, Wright AD, Collins AM, et al. Experimental human pneumococcal carriage. Journal of visualized experiments : JoVE 2013.
15. Kaiser L, Fritz RS, Straus SE, Gubareva L, Hayden FG. Symptom pathogenesis during acute influenza: interleukin-6 and other cytokine responses. Journal of medical virology 2001;64:262-268.
16. Zhao J, Yuan Q, Wang H, et al. Antibody responses to SARS-CoV-2 in patients of novel coronavirus disease 2019. Clin Infect Dis 2020.
17. To KK, Tsang OT, Leung WS, et al. Temporal profiles of viral load in posterior oropharyngeal saliva samples and serum antibody responses during infection by SARS-CoV-2: an observational cohort study. Lancet Infect Dis 2020.
18. Lin Q, Zhu L, Ni Z, Meng H, You L. Duration of serum neutralizing antibodies for SARS-CoV-2: Lessons from SARS-CoV infection. J Microbiol Immunol Infect 2020.
19. Woelfel R, Corman, V.M., Guggemos, W. Clinical presentation and virological assessment of hospitalized cases of coronavirus disease 2019 in a travel-associated transmission cluster. 2020.
20. Huang C, Wang Y, Li X, et al. Clinical features of patients infected with 2019 novel coronavirus in Wuhan, China. Lancet 2020;395:497-506.
